# Order of same-day concurrent training influences some indices of power development, but not strength, lean mass, or aerobic fitness in healthy, moderately-active men after 9 weeks of training

**DOI:** 10.1371/journal.pone.0233134

**Published:** 2020-05-14

**Authors:** Matthew J. -C. Lee, James K. Ballantyne, Javier Chagolla, William G. Hopkins, Jackson J. Fyfe, Stuart M. Phillips, David J. Bishop, Jonathan D. Bartlett

**Affiliations:** 1 Institute for Health and Sport, Victoria University, Melbourne, Australia; 2 Centre for Sport Research, School of Exercise and Nutrition Sciences, Deakin University, Australia; 3 Department of Kinesiology, McMaster University, Hamilton, Ontario, Canada; 4 School of Medicine & Health Sciences, Edith Cowan University, Joonalup, Australia; Universidade Federal de Mato Grosso do Sul, BRAZIL

## Abstract

**Background:**

The importance of concurrent exercise order for improving endurance and resistance adaptations remains unclear, particularly when sessions are performed a few hours apart. We investigated the effects of concurrent training (in alternate orders, separated by ~3 hours) on endurance and resistance training adaptations, compared to resistance-only training.

**Materials and methods:**

Twenty-nine healthy, moderately-active men (*mean ± SD*; *age* 24.5 ± 4.7 y; *body mass* 74.9 ± 10.8 kg; *height* 179.7 ± 6.5 cm) performed either resistance-only training (*RT*, *n* = 9), or same-day concurrent training whereby high-intensity interval training was performed either 3 hours before (*HIIT+RT*, *n* = 10) or after resistance training (*RT+HIIT*, *n* = 10), for 3 d^.^wk^-1^ over 9 weeks. Training-induced changes in leg press 1-repetition maximal (1-RM) strength, countermovement jump (CMJ) performance, body composition, peak oxygen uptake (${\dot{\text{V}}\text{O}}_{2\text{peak}}$), aerobic power (${\dot{\text{W}}}_{\text{peak}}$), and lactate threshold (${\dot{\text{W}}}_{\text{LT}}$) were assessed before, and after both 5 and 9 weeks of training.

**Results:**

After 9 weeks, all training groups increased leg press 1-RM (~24–28%) and total lean mass (~3-4%), with no clear differences between groups. Both concurrent groups elicited similar small-to-moderate improvements in all markers of aerobic fitness ($\dot{V}O_{2peak}$ ~8–9%; ${\dot{W}}_{LT}$ ~16-20%; ${\dot{W}}_{peak}$ ~14-15%). RT improved CMJ displacement (*mean ± SD*, 5.3 ± 6.3%), velocity (2.2 ± 2.7%), force (*absolute*: 10.1 ± 10.1%), and power (*absolute*: 9.8 ± 7.6%; *relative*: 6.0 ± 6.6%). HIIT+RT elicited comparable improvements in CMJ velocity only (2.2 ± 2.7%). Compared to RT, RT+HIIT attenuated CMJ displacement (*mean difference ± 90%CI*, -5.1 ± 4.3%), force (*absolute*: -8.2 ± 7.1%) and power (*absolute*: -6.0 ± 4.7%). Only RT+HIIT reduced absolute fat mass (*mean ± SD*, -11.0 ± 11.7%).

**Conclusions:**

In moderately-active males, concurrent training, regardless of the exercise order, presents a viable strategy to improve lower-body maximal strength and total lean mass comparably to resistance-only training, whilst also improving indices of aerobic fitness. However, improvements in CMJ displacement, force, and power were attenuated when RT was performed before HIIT, and as such, exercise order may be an important consideration when designing training programs in which the goal is to improve lower-body power.

## Introduction

Concurrent performance of endurance and resistance exercise within a periodised training program may benefit athletic performance, by concomitantly improving aerobic fitness, muscle strength, and power [1]. However, compared to resistance-only training, concurrent training may compromise strength, power, and/or hypertrophic adaptations, which is commonly referred to as the *“interference effect”* [1, 2]. Nonetheless, such interference is not consistently observed, as comparable improvements in muscle strength and hypertrophy have also been reported following concurrent versus resistance-only training [3–7]. The potential for, and the degree of, interference may relate to the chosen dependent variables and performance tests [8], as well as the manipulation of training variables (e.g., exercise order, between-mode recovery duration, exercise mode, frequency, intensity, volume [1, 9, 10]), and ‘*non-training*’ variables (e.g., participant training status and nutrient availability [8, 11]).

Residual fatigue and substrate depletion can reduce the quality and performance of a subsequent training session [12]. Both endurance [13] and resistance exercise performance [14–16] can be impaired when preceded in the same session by the ‘contrasting’ exercise mode, compromising the work done, and potentially the adaptive training stimulus [17, 18]. In support of this, prioritising resistance exercise before endurance exercise sessions has been shown in some studies to induce superior lower-body strength, muscle function, and neuromuscular adaptations [19–21]. Conversely, performing endurance before resistance training may favour greater improvements in submaximal [22] and maximal aerobic capacity [23], and endurance performance [23, 24]. Thus, adaptations to same-session concurrent training may be order-dependent and dictated by the first exercise mode performed. This conclusion was broadly supported by two recent meta-analyses [25, 26], at least for maximal dynamic strength development. However, no order effect was evident for aerobic capacity, static strength, hypertrophy, and body fat percentage [26]. Of note, an important omission from many studies investigating the effect of concurrent exercise order is the absence of a resistance-only training group [19–22, 27–33], which prevents inferences being made about whether resistance training adaptations were compromised by the addition of endurance training *per se*, regardless of whether one exercise order induced superior adaptations.

Concurrent training studies often schedule sessions back-to-back (which may be sub-optimal for adaptations, despite the time-efficiency) [34–36], or on separate days (allowing longer between-mode recovery) [36–39]. However, in many applied sport settings, concurrent sessions are separated by a few hours of recovery [40–43], which may minimise the potential antagonism between transient molecular-level responses that respectively promote endurance and resistance adaptations [44]. Despite such practices being used in the field, few studies to date have adopted such recovery durations. We previously demonstrated in recreationally-active men that 8 weeks of high-intensity interval training (HIIT) performed 10 min before resistance-only training interfered with improvements in lower-body strength and lean mass, as well as countermovement jump force and power [35]. Since HIIT is relevant to many athletic populations [45], the question remains whether the attenuated adaptations could be mitigated by altering the exercise order, and providing a recovery window that better mimics applied sport training program designs.

Thus, to address previous gaps in the literature, we compared adaptations to concurrent training in varying orders to resistance-only training. Specifically, we investigated the impact of concurrent training and exercise order on the development of strength, lean mass, power, as well as aerobic fitness, over nine weeks in healthy, moderately-active men. We also sought to employ a more ecologically-valid recovery period between concurrent sessions (3 hours), as is often utilised in the field. We hypothesised that compared to resistance-only training, concurrent training would attenuate the improvements in resistance training adaptations; however, performance of endurance or resistance exercise first would benefit that respective exercise mode-specific adaptation.

## Materials and methods

### Participants

**Fig 1:**
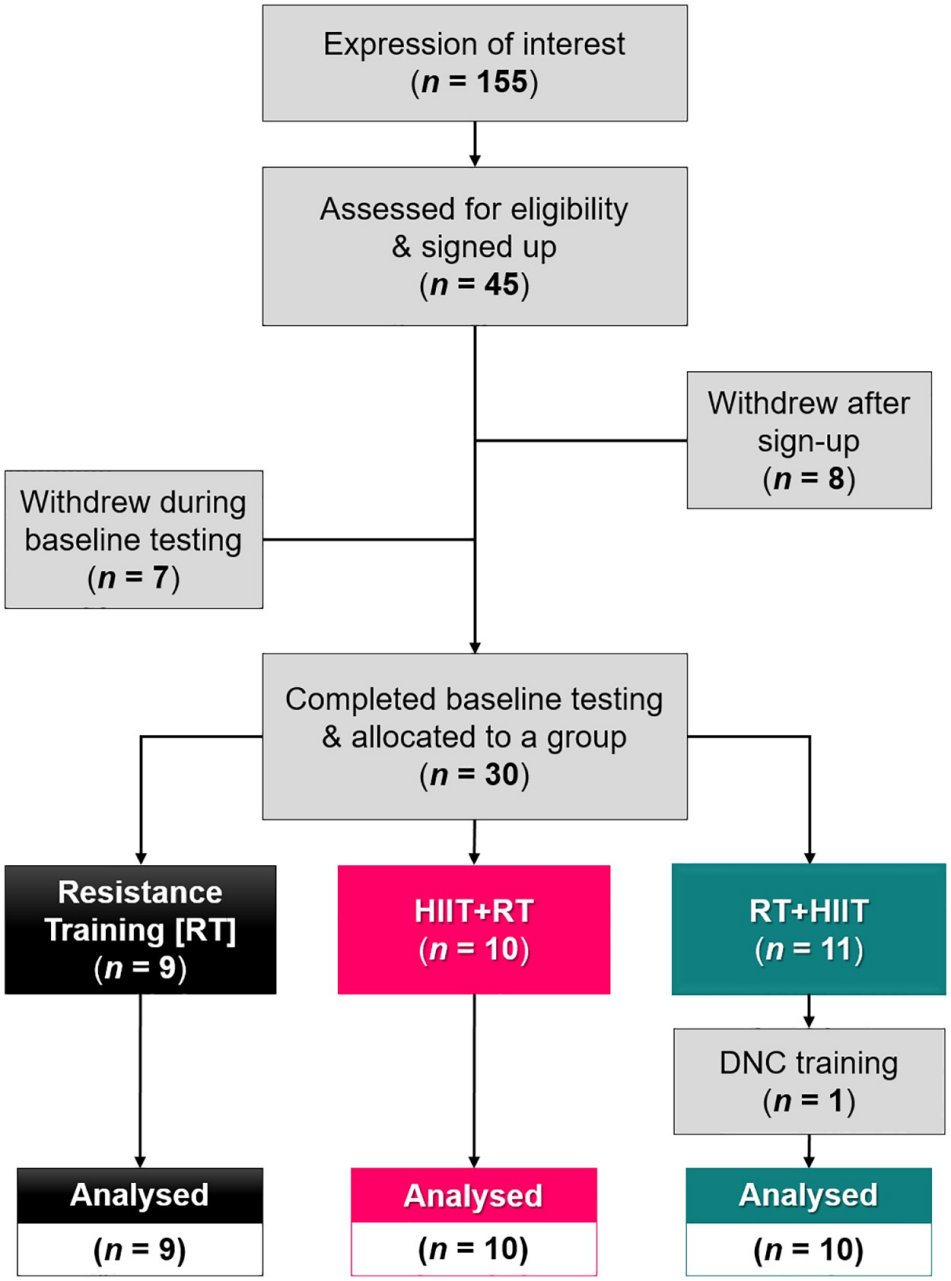
Flow chart depicting the recruitment process leading to the final training group sample sizes. Out of 155 initial expressions of interest, forty-five healthy, moderately-active men met the inclusion criteria and volunteered to participate in the study. During the preliminary testing phase, fifteen participants withdrew for various reasons (e.g. changes in availability, time commitment, and for personal reasons). Thirty participants completed the baseline testing and were allocated to one of the three training groups. During the training phase, one participant withdrew due to an injury sustained outside of the study. Therefore, twenty-nine participants completed the training study and were included in the final analyses. *HIIT* high-intensity internal training; *DNC* did not complete.

Twenty-nine healthy, moderately-active men completed this study (*mean ± SD*; *age* 24.5 ± 4.7 y; *body mass* 74.9 ± 10.8 kg; *height* 179.7 ± 6.5 cm; Fig 1). Participants were non-smokers, free from any pre-existing medical conditions and musculoskeletal injuries, were habitually exercising for ≥ 30 minutes, 2–3 d^.^wk^-1^, but not following a prescribed training program. During the initial consultation, participants were asked to self-report the intensity, frequency, duration, and types of physical activities habitually completed during the previous 3 months. Based on these self-reports, the participants were exercising ~3.1 ± 1.3 sessions per week, ~64 ± 21 min/session. Typical activities listed included “resistance exercise” (i.e., “gym”-based training, “weights”), “endurance exercises” (“running”, “cycling”, “swimming”), and various sports (“cricket”, “Australian Rules football”, “soccer”, “basketball”, “tennis”, “volleyball”). Participants were given comprehensive, written and verbal information about the study before providing written consent. This research was performed in accordance with the Declaration of Helsinki, and all procedures were approved by the Victoria University Human Research Ethics Committee (HRE15-292).

### Experimental design overview

**Fig 2:**
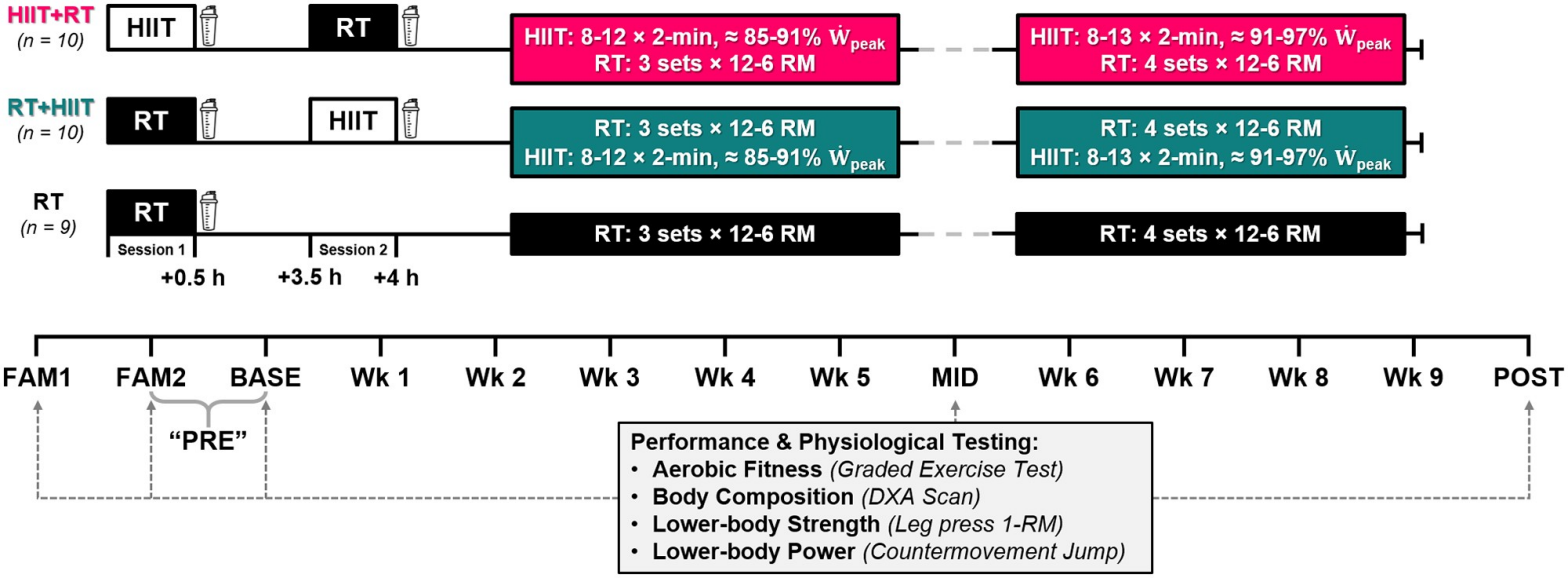
Schematic overview of the study. *HIIT* high-intensity interval training; *RT* resistance training only; *HIIT+RT* high-intensity interval training and resistance training group; *RT+HIIT* resistance and high-intensity interval training group; 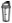 whey protein (0.25 g^.^kg_BM_^-1^); *DXA* dual-energy x-ray absorptiometry; *1-RM* one repetition maximum; ${\dot{W}}_{peak}$ peak aerobic power; *FAM* familiarisation trial; *BASE* baseline trial; “*PRE”* mean of FAM2 and BASE trials; *MID* mid-training trial; *POST* post-training trial.

Participants completed two familiarisation trials (*FAM1* & *FAM2*) before baseline (*BASE*) assessments of leg press 1-repetition maximum strength (1-RM), countermovement jump (CMJ) performance, body composition, and aerobic fitness; these assessments were repeated after 5 (*MID*) and 9 (*POST*) weeks of training (Fig 2). Participants were ranked according to baseline levels of strength, ${\dot{\text{V}}\text{O}}_{2\text{peak}}$, and total lean mass (Table 1), and then allocated into one of three training groups: 1) *HIIT+RT*, high-intensity interval training before resistance training (*n* = 10); 2) *RT+HIIT*, resistance before high-intensity interval training (*n* = 10); or 3) *RT*, resistance training only (*n* = 9). Participants were allocated via minimisation to standardise baseline 1-RM strength, total lean mass, and aerobic fitness between groups. Effect size analysis revealed some small differences between groups at baseline (i.e., standardised effect size 0.2–0.6; Table 1); these were accounted for prior to analysis. Participants trained 3 d^.^wk^-1^ for 9 weeks; the same-day concurrent sessions were separated by 3 hours of recovery (*mean ± SD*, HIIT+RT 3.1 ± 0.2 h; RT+HIIT 3.1 ± 0.5 h).

**Table 1:** Baseline characteristics of each training group. Data are mean ± SD.

	RT-only *(n = 9)*	HIIT+RT *(n = 10)*	RT+HIIT *(n = 10)*
**Lower-Body Maximal Strength**			
Leg press 1-RM (kg)	344 ± 100	329 ± 94	327 ± 90
Relative 1-RM (kg^.^kg_BM_^-1^)	4.5 ± 0.9	4.4 ± 0.9	4.4 ± 0.9
**Countermovement Jump Performance**			
Peak Displacement (cm)	36 ± 7.0	35 ± 4.2	36 ± 5.9
Peak Velocity (m^.^s^-1^)	2.72 ± 0.24	2.71 ± 0.15	2.75 ± 0.20
Peak Force (N)	959 ± 227 ^□^^△^	1037 ± 256	1014 ± 174
Relative Peak Force (N^.^kg_BM_^-1^)	13 ± 2.4 ^□^^△^	14 ± 2.0	14 ± 2.7
Peak Power (W)	3814 ± 689	3783 ± 720	3786 ± 712
Relative Peak Power (W^.^kg_BM_^-1^)	51 ± 9.5	51 ± 4.8	51 ± 7.9
**Body Composition**			
Total Lean Mass [*LM*] (kg)	54 ± 6.7	53 ± 8.1 ^○^^△^	54 ± 8.1
*Upper body LM (kg)*	35 ± 4.0	33 ± 5.2 ^○^^△^	35 ± 4.8
*Lower body LM (kg)*	19 ± 2.9	20 ± 3.1	20 ± 3.4
Fat Mass (kg)	14 ± 5.9	15 ± 6.8 ^△^	13 ± 5.9
**Aerobic Fitness**			
Absolute ${\dot{\text{V}}\text{O}}_{2\text{peak}}$ (L^.^min^-1^)	3.33 ± 0.76	3.36 ± 0.62	3.24 ± 0.54
Relative ${\dot{\text{V}}\text{O}}_{2\text{peak}}$ (mL^.^kg^.^min^-1^)	44 ± 8.1	45 ± 7.2	44 ± 5.9
Lactate Threshold [${\dot{\text{W}}}_{\text{LT}}$] (W)	157 ± 45	162 ± 38 ^△^	153 ± 31
Peak Aerobic Power [${\dot{\text{W}}}_{\text{peak}}$] (W)	215 ± 54	217 ± 46 ^△^	205 ± 41
**Habitual Dietary Intake**			
Energy (kcal^.^kg^BM.^d^-1^)	33 ± 8 ^□^^△^	36 ± 11	36 ± 9
Carbohydrate (g^.^kg^BM.^d^-1^)	3.2 ± 1.3 ^□^^△^	4.0 ± 1.9	3.6 ± 1.0
Protein (g^.^kg^BM.^d^-1^)	2.0 ± 0.7 ^□^^△^	1.9 ± 0.6 ^△^	2.1 ± 0.6
Fat (g^.^kg^BM.^d^-1^)	1.2 ± 0.4 ^□^^△^	1.5 ± 0.4 ^△^	1.4 ± 0.4

○small difference vs RT;

^□^ small difference vs HIIT+RT;

^△^ small difference vs RT+HIIT. (i.e., standardised effect size, ES = 0.20–0.60). Differences at baseline were accounted for in the subsequent statistical analyses. *kg*_*BM*_^*-1*^, per kilogram of body mass

### Familiarisation & baseline testing

Each visit was separated by ≥ 24 hours of recovery. Test times were standardised for each participant and replicated on subsequent visits. Participants self-reported abstinence from exercise, as well as alcohol and caffeine consumption, for at least 24 hours before all procedures. Reliability data, determined using the FAM2 and BASE trials, are provided for all performance tests in S1 Appendix.

### Lower-body power & strength: CMJ & leg press 1-RM tests

Upon arrival, participants completed a standardised warm up (10 sub-maximal repetitions of leg press, glute bridges, and unloaded body-weight squats, respectively). The CMJ test was performed on a commercially-available force plate (400S, Fitness Technology, Adelaide, Australia). After completing three submaximal jumps (50%, 75%, and 90% effort), participants performed three maximal jumps, each separated by a 1-minute recovery [35]. Arm-swing was minimised by holding a light wooden pole (< 0.5 kg) across the shoulders. The raw force-time data (sampled at 600 Hz) was collected using Ballistic Measurement System software (Fitness Technology, Adelaide, Australia), then exported to and analysed in Microsoft Excel using a spreadsheet specifically-formulated for analysing CMJ data to derive peak displacement, velocity, force, and power [46]. For each variable, the average of the three maximal attempts was used for analysis.

During the same visit, after 5 minutes of passive rest, the 1-RM test was performed on a plate-loaded 45° incline leg press (Hammer Strength Linear, Schiller Park, IL, USA). The leg press sled weighed 53 kg, which was incorporated into the calculation of the total load lifted. Participants performed 3 warm-up sets (5×50%, 3×70%, and 1×90% of estimated 1-RM) before attempting ≤5 single repetitions, until a successful 1-RM was determined. All sets were separated by a 2-minute rest. Failure was determined when the load could not be lifted through the required range, from full knee extension through 90° knee flexion; this was monitored by the investigators and confirmed using video footage. A 1-RM prediction table [47] and 10-point resistance exercise-specific RPE scale (Repetitions-in-Reserve, *RIR*, [48]) were used to aid load progression between attempts.

### Aerobic fitness: Graded exercise test (GXT) and verification bout (VB)

During a separate visit, peak oxygen uptake (${\dot{\text{V}}\text{O}}_{2\text{peak}}$), lactate threshold (${\dot{\text{W}}}_{\text{LT}}$), and peak aerobic power (${\dot{\text{W}}}_{\text{peak}}$) were determined via a GXT and a supramaximal, constant-workload VB, both performed on an electromagnetically-braked cycle ergometer (Lode Excalibur Sport, Groningen, The Netherlands). The GXT protocol (described in [49]) involved multiple 4-minute stages of incremental workloads performed at 70 ± 10 RPM. Using a 20-gauge cannula, antecubital blood (~1 mL) was sampled at rest and during the last 15 seconds of each stage, and immediately analysed in duplicate for lactate concentration (YSI 2300 STAT Plus; YSI Inc., Yellow Springs, OH, USA). ${\dot{\text{W}}}_{\text{LT}}$ was defined by the modified Dmax method using Lactate-E [50]. Heart rate (Polar FT1, Kempele, Finland) and rating of perceived exertion (RPE) [51] were recorded in the last 10 seconds of each stage. After a 5-minute recovery, participants performed the VB, at 105% of the GXT-derived ${\dot{\text{W}}}_{\text{peak}}$, maintaining a high cadence (90–100 RPM) until exhaustion. Consistent verbal encouragement was given throughout. Both the GXT and VB were terminated volitionally by the participant, or when a cadence > 60 RPM could not be maintained. Expired gases were sampled automatically every 15 seconds throughout (Moxus Modular ${\dot{\text{V}}\text{O}}_{2}$ System, AEI Technologies, Pittsburgh, PA, USA); ${\dot{\text{V}}\text{O}}_{2\text{peak}}$ was averaged between the two highest consecutive 15-second values. Before each test, the gas analysers and flowmeter were calibrated against known concentrations and volumes, respectively [35].

### Body composition: Dual-energy X-ray Absorptiometry (DXA)

Body composition was estimated via whole-body DXA (GE Lunar iDXA, GE Lunar Corp, Madison, WI, USA). Before each scan, the DXA was calibrated as per the manufacturer’s guidelines. Each participant’s scan was conducted and analysed by the same certified technician. Participants arrived after an overnight fast and bladder void, and having refrained from exercise 24 hours prior [52, 53]. DXA scans were analysed using the software-defined regions of interest (enCORE, version 16, GE Medical Systems, Madison, WI, USA). The measured regions included the left and right arms and legs, and the trunk; the head was excluded, as recommended by Kruger *et al*. [54]. Two scans were conducted at baseline, on separate days, to assess between-day reliability (S1 Appendix).

### Exercise training

The training programs were modified from previously-published training programs from our laboratory [35]; full details are provided in S2 Appendix. Before every session, to monitor “readiness-to-train”, participants reported their perceived levels of fatigue, sleep quality, general muscle soreness, stress, and mood, from 1 (worst) to 5 (best), with increments of 0.5 [55] (S3 Appendix). After each session, participants rated their perceived exertion (sRPE) using the CR1-10 scale [56] (S3 Appendix). Internal total load was calculated for each session by multiplying sRPE by the session duration; this is a valid and reliable method for monitoring internal training load for both aerobic and resistance training [57].

#### Resistance training (RT)

The intensity and volume progressed from 3–4 sets of 12–6 repetitions, with 2 minutes of rest between sets. A warm-up set (5 reps at ~75% of the prescribed training load) was performed before the first 2 exercises. Excluding leg press (for which 1-RM was directly measured), all other exercises were prescribed according to the maximum number of repetitions possible for a given load (i.e., *n*-RM). Training loads were adjusted according to the prescribed changes in *n*-RM. A 1-RM prediction table [47], and participant feedback using the 10-point RIR scale [48] were used as accessory tools to aid load prescription (S3 Appendix).

#### High-intensity interval training (HIIT)

On an electromagnetically-braked cycle ergometer (Velotron, Racer-Mate, Seattle, WA, USA) participants completed a standardised warm-up (5 minutes at 75 W, ~70 RPM), followed by multiple 2-minute cycling intervals at 90-100 RPM, each separated by 1-minute recovery periods. Progressive overload was achieved by modifying the volume (8–13 intervals) and intensity (40–90% of the difference between ${\dot{\text{W}}}_{\text{LT}}$ and ${\dot{\text{W}}}_{\text{peak}}$, corresponding to ≈ 85-97% ${\dot{\text{W}}}_{\text{peak}}$) in each session. After the *MID* GXT, the intensity for the subsequent sessions in weeks 6 to 9 was adjusted using the updated ${\dot{\text{W}}}_{\text{LT}}$ and ${\dot{\text{W}}}_{\text{peak}}$ data.

### Diet and exercise control

Participants were instructed to maintain their habitual physical activity levels throughout the study, which was regularly monitored via self-reported diaries. Any additional, non-prescribed exercise was documented using an online training diary, in which the session type, duration, and sRPE were recorded. Habitual dietary intake was recorded at baseline using 3-day food diaries (including one weekend day), which were analysed using an online nutrition tracking application (https://cronometer.com/). Effect size analysis revealed trivial-to-small differences between groups for habitual energy and macronutrient intake (ranging from -0.06 to 0.55; Table 1). Whey protein isolate (0.25 g^.^kg_BM_^-1^, BodyScience, QLD, Australia) was provided immediately after each session; this dose has been shown to be appropriate for maximising post-exercise muscle protein synthesis [58].

### Statistical analyses

All variables (excluding the “readiness-to-train” data) were log-transformed before analysis [59]. To improve the precision of the estimate, the mean of the *FAM2* and *BASE* trials represented each participant’s pre-training value (i.e., *PRE*; Fig 2). Change scores from *PRE* to *MID*, and *PRE* and *POST* training respectively were analysed using a mixed model (*Proc Mixed* in SAS Studio University Edition, v9.4, SAS Institute, Cary, NC). Fixed effects were the interaction of training group (*HIIT+RT*, *RT+HIIT*, *RT*) with time (*MID* and *POST-training*), plus the interaction of group with *PRE* values (linear numeric, to adjust for between-group differences from the combined pre-training mean of all participants). Random effects specified different residual variances (of the change scores) at *MID* and *POST* in each group, allowing for correlations between the *MID* and *POST* residuals within each group. The residual variances were back-transformed to give standard deviations of the change scores in percent units. “Readiness-to-train” and training load data were analysed with a reliability mixed model; fixed effects accounted for session and weekly means, and random effects accounted for within-subject variability within and between weeks, in each of the three groups.

The magnitudes of all effects were assessed using standardised effect sizes (ES), where thresholds representing trivial, small, and moderate effects were respectively given by < 0.2, 0.2–0.6, and 0.6–1.2 times the combined between-subject baseline standard deviation (SD) [59]. For “readiness-to-train” and training load data, the standardising SD was derived in the mixed model from the random effects for a typical session.

Uncertainty in the estimates of effects are presented as 90% compatibility intervals (90%CI). Decisions about magnitudes accounting for the uncertainty were based on one-sided hypothesis tests [60]. The *p* value for rejecting an hypothesis of a given substantial magnitude was the area of the sampling *t* distribution of the effect statistic with values of that magnitude [60]. For comparisons of each concurrent group with the resistance-only group (i.e., clinically or practically relevant effects which could result in a treatment being implemented), hypotheses of harm (*p*^*H*^) and benefit (*p*^*B*^) were respectively rejected if *p*^*H*^<0.005 and *p*^*B*^<0.25 (strong and weak rejection, respectively). For all other effects (including comparisons between the concurrent groups, and analyses of “readiness-to-train” and training load data), hypotheses of a substantial negative (*p*^*–*^) and positive effect (*p*^+^) were rejected if their respective *p* values were < 0.05 (moderate rejection); if both hypotheses were rejected, effects were described as clearly trivial. If only one hypothesis was rejected, the *p* value for the other hypothesis (which corresponds to the posterior probability of the magnitude of the true effect in a reference Bayesian analysis with a minimally informative prior [61, 62]), was reported using the following scale: *p* = 0.25-0.75, “*possibly*”; *p* = 0.75–0.95, “*likely*”; *p* = 0.95–0.995, “*very likely*”; *p*>0.995, “*most likely*” [59]. This scale was also used to interpret clearly trivial effects, which is given by the area of the sampling distribution in trivial values. If neither hypothesis was rejected, the effect was described as *unclear*, and the effect size is reported without a probabilistic qualifier. Only effects which are “*likely*”, or greater, are considered meaningful.

To account for inflation of error arising from multiple inferences, effects were considered decisive with more conservative thresholds (*p*^*H*^<0.001; *p*^*B*^<0.05, *p*^*–/+*^<0.005) and are formatted **bold** in tables, and with capitalised letters in figures. Precise *p* values (unless <0.001) from the one-sided hypothesis tests, plus traditional NHST *p* values, are provided in S4 Appendix.

## Results

### Countermovement jump performance

**Fig 3:**
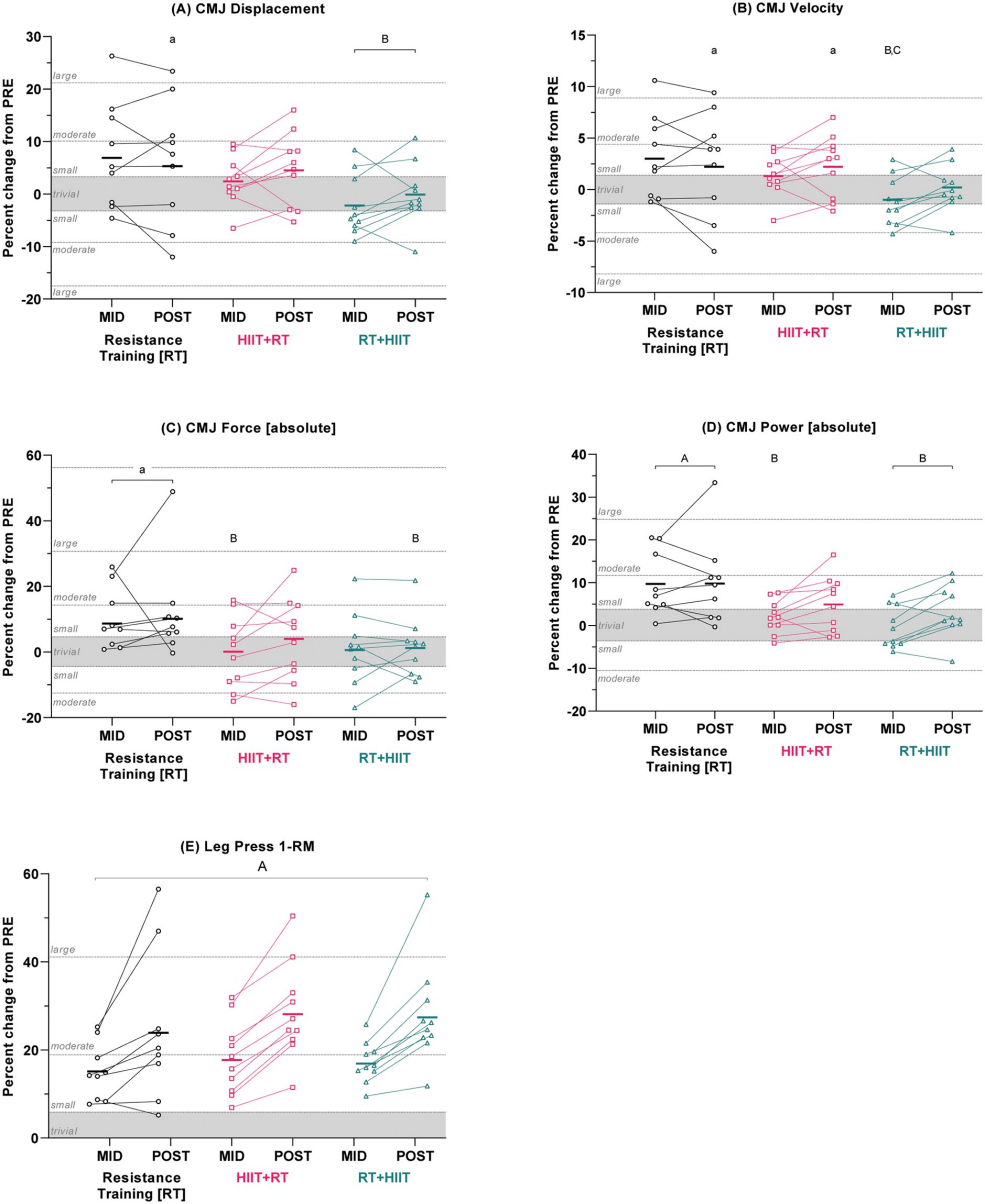
Percent changes in strength and power indices after 5 (MID) and 9 (POST) weeks of training. (A) Peak CMJ displacement, (B) velocity, (C) force, (D) power, and (E) leg press 1-RM strength. Data are adjusted group means (solid dash), plus unadjusted individual percent changes. The trivial region was determined from the smallest worthwhile change for each variable (0.2 × combined between-subject baseline SD). a/A = within-group difference vs PRE; b/B = between-group difference vs RT at same time point; c/C = between-group difference vs HIIT+RT at same time point. Capital letters denote effects that remained clear after adjustment for multiple comparisons.

Five weeks of RT improved peak force ([*ES ±90%CI] absolute*: 0.37 ±0.28) and power (*absolute*: 0.50 ±0.21; *relative*: 0.42 ±0.25); any changes in either concurrent training group were either trivial, not meaningful (i.e., only “*possibly*” different from PRE), or unclear. Compared to RT, RT+HIIT attenuated improvements in peak displacement (-0.55 ±0.42), velocity (-0.57 ±0.42), and power (*absolute*: -0.53 ±0.24; *relative*: -0.54 ±0.32). Similarly, compared to RT, HIIT+RT attenuated improvements in force (*absolute*: -0.37 ±0.37) and power (*absolute*: -0.40 ±0.22; *relative*: -0.35 ±0.29). There was also an exercise order effect for velocity, favouring HIIT+RT (0.33 ±0.23). After 9 weeks, RT improved all CMJ variables, with the exception of relative force (*displacement*: 0.32 ±0.26; *velocity*: 0.31 ±0.25; *absolute force*: 0.43 ±0.29; *absolute power*: 0.50 ±0.26; *relative power*: 0.39 ±0.28). Similar to RT, HIIT+RT improved peak velocity only (0.31 ±0.23); all other changes for both concurrent groups were either trivial, not meaningful, or unclear. Compared to RT, RT+HIIT attenuated improvements in peak displacement (-0.33 ±0.29), absolute force (-0.38 ±0.35), and absolute power (-0.33 ±0.28) (Fig 3a–3d and Table 2). For most variables, the standard deviations (SD) of the within-group change scores PRE-to-POST were similar to, or less than, the SD of the change scores from the reliability study (i.e., $\sqrt{2}$×CV; S1 Appendix), with the exception of absolute power, for which there was evidence of individual responses in RT and HIIT+RT (RT 7.6%; HIIT+RT 5.8%; vs 4.9%).

**Table 2:** Between-group comparisons of changes in all performance measures, from *PRE*-to-*MID* and *PRE*-to-*POST* training. Data are mean percent differences and 90% compatibility intervals (±90%CI), plus magnitude-based decisions of standardised effect sizes (ES) and qualitative likelihoods†.

	PRE to MID			PRE to POST		
	*“Interference effect”*		*“Order effect”*	*“Interference effect”*		*“Order effect”*
	RT vs HIIT+RT	RT vs RT+HIIT	HIIT+RT vs RT+HIIT	RT vs HIIT+RT	RT vs RT+HIIT	HIIT+RT vs RT+HIIT
**Lower-body Maximal Strength**						
Leg Press 1-RM	2.2, ±4.7%	1.6, ±3.3%	-0.6, ±4.2%	3.4, ±8.5%	2.8, ±8.4%	-0.6, ±6.0%
	*Trivial*, ^●^↔	***Trivial***, ^●^↔	*Trivial*, ^●^↔	*Trivial*	*Trivial*	*Trivial*, ^●^↔
**Countermovement Jump Performance**						
Peak CMJ Displacement	-4.2, ±6.3%	-8.4, ±6.0%	-4.4, ±3.4%	-0.8, ±5.0%	-5.1, ±4.3%	-4.3, ±4.1%
	***Small***, °↓	***Small***, ^●^↓	***Small***, °↓	*Trivial*, °↔	***Small***, ^●^↓	***Small***, °↓
Peak CMJ Velocity	-1.7, ±2.8%	-3.9, ±2.8%	-2.3, ±1.5%	0.0, ±2.2%	-2.0, ±1.9%	-2.0, ±1.8%
	***Small***, °↓	***Small***, ^●^↓	***Small***, ^●^↓	*Trivial*, °↔	***Small***, °↓	***Small***, °↓
Peak CMJ Force *(absolute)*	-8.0, ±7.4%	-7.4, ±7.6%	0.5, ±8.5%	-5.6, ±7.4%	-8.2, ±7.1%	-2.7, ±7.0%
	***Small***, ^●^↓	***Small***, °↓	*Trivial*	***Small***, °↓	***Small***, ^●^↓	*Trivial*, °↓
Peak CMJ Power *(absolute)*	-7.1, ±3.5%	-9.4, ±3.5%	-2.5, ±2.9%	-4.4, ±5.1%	-6.0, ±4.7%	-1.6, ±3.9%
	***Small***, ^●^↓	***Small***, ^●^↓	***Trivial***, °↓	***Small***, °↓	***Small***, ^●^↓	*Trivial*, ^●^↔
**Body Composition**						
Total Lean Mass	0.1, ±1.6%	0.4, ±1.8%	0.3, ±1.4%	0.1, ±1.6%	1.0, ±1.6%	0.9, ±1.1%
	*Trivial*, ^●^↔	*Trivial*, ^●^↔	***Trivial***, ^●^↔	*Trivial*, ^●^↔	***Trivial***, ^●^↔	***Trivial***, ^●^↔
*Upper-Body Lean Mass*	0.7, ±1.4%	0.6, ±1.7%	-0.1, ±1.4%	0.5, ±1.8%	1.8, ±1.5%	1.3, ±1.6%
	***Trivial***, ^●^↔	*Trivial*, ^●^↔	***Trivial***, ^●^↔	*Trivial*, ^●^↔	***Trivial***, ^●^↔	***Trivial***, ^●^↔
*Lower-Body Lean Mass*	-0.6, ±2.4%	0.4, ±2.9%	0.9, ±2.3%	-0.3, ±2.2%	-0.2, ±2.3%	0.1, ±1.7%
	***Trivial***, ^●^↔	*Trivial*, ^●^↔	***Trivial***, ^●^↔	***Trivial***, ^●^↔	***Trivial***, ^●^↔	***Trivial***, ^●^↔
Total Fat Mass	-3.9, ±5.2%	-8.9, ±4.5%	-5.2, ±6.0%	-4.9, ±6.5%	-12.8, ±6.3%	-8.3, ±7.7%
	***Trivial***, ^●^↔	***Small***, °↑	***Trivial***, ^●^↔	***Trivial***, ^●^↔	***Small***, ^●^↑	***Small***, °↑
**Aerobic Fitness**						
$\dot{V}{\text{O}}_{2\text{peak}}$ *(relative)*	7.9, ±4.1%	8.0, ±5.2%	0.1, ±4.9%	11.0, ±3.5%	10.0, ±3.7%	-0.9, ±2.9%
	***Small***, ^●^↑	***Small***, ^●^↑	*Trivial*	***Moderate***, ^●^↑	***Small***, ^●^↑	*Trivial*, ^●^
Lactate Threshold	15.2, ±8.9%	9.9, ±9.1%	-4.6, ±4.8%	22.3, ±12.4%	18.7, ±13.6%	-3.0, ±7.2%
	***Moderate***, ^●^↑	*Small* ^●^↑	***Small***, °↓	***Moderate***, ^●^↑	*Moderate*, ^●^↑	*Trivial*, °↓
Peak Aerobic Power	11.0, ±6.2%	9.8, ±5.2%	-1.0, ±4.9%	15.7, ±6.2%	15.1, ±6.4%	-0.5, ±5.5%
	***Small***, ^●^↑	***Small***, ^●^↑	*Trivial*, ^●^	***Moderate***, ^●^↑	***Moderate***, ^●^↑	*Trivial*

^†^ < 0.20 = trivial; 0.20–0.60 = small; 0.60–1.2 = moderate. 0.20 defined smallest important effects.

°possible effect (i.e. p = 0.25–0.75);

^●^likely or greater effect (i.e., p ≥ 0.75); “unclear” comparisons have no symbol. Effects that remained clear after adjustment for multiple inferences are in **bold**.

^↓^ harmful/negative;

^↑^ beneficial/positive;

^↔^ trivial.

### Leg press 1-RM strength

All groups increased absolute leg press 1-RM after 5 weeks (*[ES ±90%CI]* RT 0.49 ±0.14; HIIT+RT 0.56 ±0.18; RT+HIIT 0.54 ±0.14) and after 9 weeks (RT 0.74 ±0.29; HIIT+RT 0.86 ±0.24; RT+HIIT 0.84 ±0.24), with no between-group differences for the changes at either time-point (Fig 3e and Table 2). Similar effects were observed when 1-RM was expressed relative to body mass (kg^.^kg_BM_^-1^), see S4 Appendix. The SD of the change scores PRE-to-POST (RT 12.4%, HIIT+RT 8.3%, RT+HIIT 7.9%, respectively) were considerably larger than the SD of the change scores in the reliability study (i.e., $\sqrt{2}$×CV = 4.9%; S1 Appendix), representing evidence of individual responses in each group.

### Body composition

**Fig 4:**
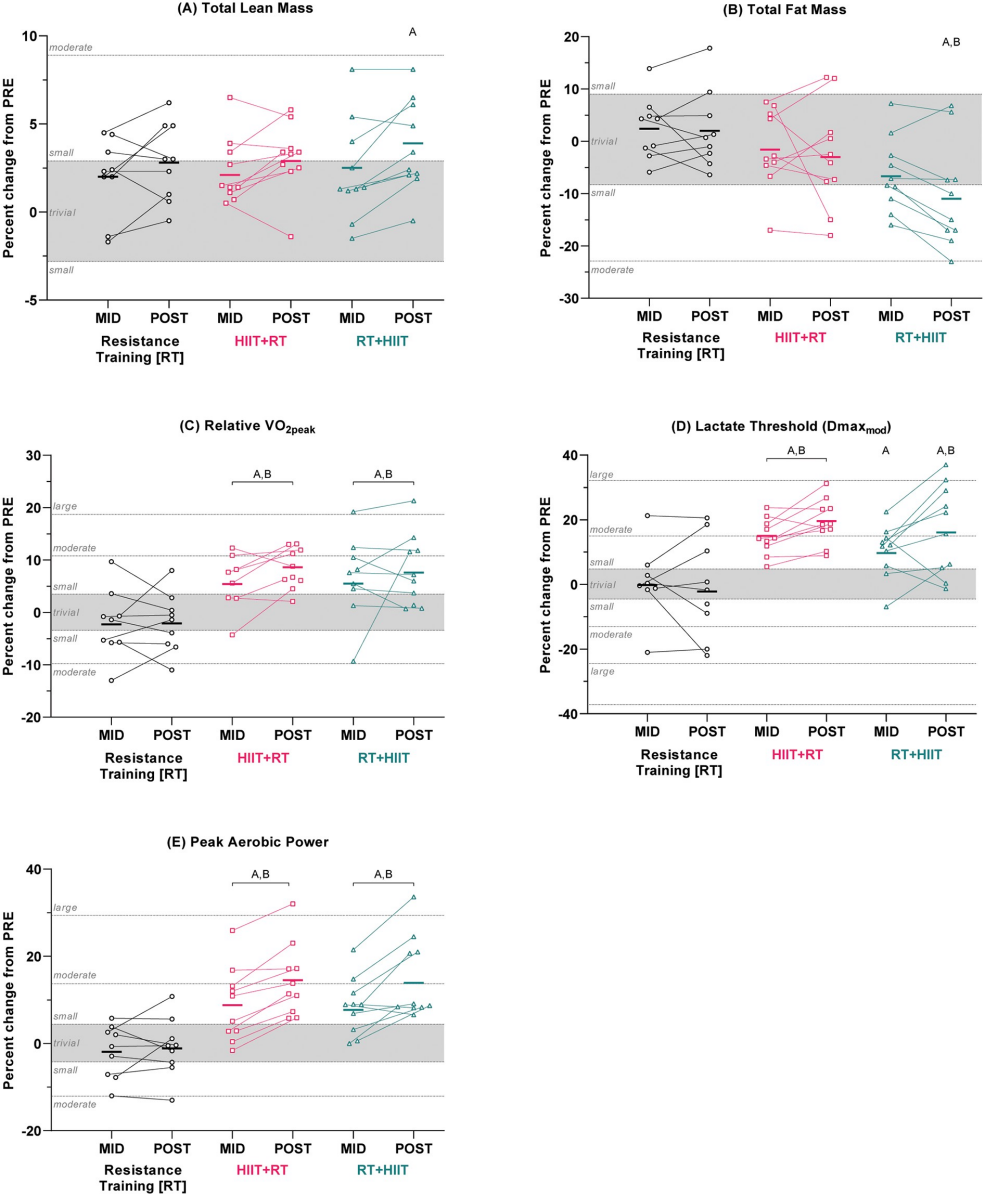
Percent changes in body composition and aerobic fitness after 5 (MID) and 9 (POST) weeks of training. (A) Total lean mass, (B) total fat mass, (C) relative $\dot{V}{\text{O}}_{2}\text{peak}$, (D) lactate threshold, and (E) peak aerobic power. Data are adjusted group means (solid dash) with unadjusted individual percent changes. The trivial region was determined from the smallest worthwhile change for each variable (0.2 × combined between-subject baseline SD). a/A = within-group difference vs PRE; b/B = between-group difference vs RT at same time point; c/C = between-group difference vs HIIT+RT at same time point. Capital letters denote effects that remained clear after adjustment for multiple comparisons. *N.B. due to an equipment fault at the time of testing, PRE-to-MID changes are unavailable for 2 participants in HIIT+RT ($\dot{V}O_{2}peak$), and one from RT (${\dot{W}}_{LT}$).*

After 5 weeks, any changes in total, upper-, and lower-body lean mass, as well as total fat mass, were either clearly trivial, or not meaningful. Likewise, any differences between groups were trivial or not substantial. After 9 weeks of training, only RT+HIIT induced small improvements in total lean mass ([*ES ±90%CI*] 0.26 ±0.08), upper body lean mass (0.29 ±0.09; S1 Fig), and total fat mass (-0.27 ±0.16). All other within-group changes were either clearly trivial, or not meaningful. However, all between-group differences were clearly trivial, with the exception of total fat mass, which was reduced more by RT+HIIT than RT (-0.32 ±0.18) (Fig 4a and 4b, and Table 2). Comparing the SDs of the changes PRE-to-POST with the SD of the changes in the reliability study (S1 Appendix), individual responses were evident in some groups for total lean mass (RT 2.3% vs 1.3%), upper body lean mass (HIIT+RT 2.4% vs 2.1%), lower body lean mass (RT 3.2%, HIIT+RT 2.1%, RT+HIIT 2.4%, vs 1.6%) and total fat mass (RT 6.3%, HIIT+RT 11.0%, RT+HIIT 11.7%, vs 4.1%).

### Aerobic fitness

After 5 weeks, concurrent training, regardless of exercise order, improved ${\dot{\text{V}}\text{O}}_{2\text{peak}}$ ([*ES ±90%CI*] HIIT+RT 0.31 ±0.18; RT+HIIT 0.31 ±0.25), ${\dot{\text{W}}}_{\text{LT}}$ (HIIT+RT 0.60 ±0.17; RT+HIIT 0.40 ±0.21), and ${\dot{\text{W}}}_{\text{peak}}$ (HIIT+RT 0.39 ±0.21; RT+HIIT 0.35 ±0.15). These changes were superior to RT (${\dot{\text{V}}\text{O}}_{2\text{peak}}$: RTvsHIIT+RT 0.44 ±0.24; RTvsRT+HIIT 0.45 ±0.29; ${\dot{\text{W}}}_{\text{LT}}$: RTvsHIIT+RT: 0.61 ±0.35; ${\dot{\text{W}}}_{\text{peak}}$: RTvsHIIT+RT: 0.49 ±0.28; RTvsRT+HIIT: 0.44 ±0.24). Likewise, after 9 weeks, both concurrent groups similarly improved ${\dot{\text{V}}\text{O}}_{2\text{peak}}$ (HIIT+RT 0.48 ±0.15; RT+HIIT 0.43 ±0.17), ${\dot{\text{W}}}_{\text{LT}}$ (HIIT+RT 0.77 ±0.21; RT+HIIT 0.64 ±0.33) and ${\dot{\text{W}}}_{\text{peak}}$ (HIIT+RT 0.63 ±0.23; RT+HIIT 0.61 ±0.23). Again, these changes were superior to RT (${\dot{\text{V}}\text{O}}_{2\text{peak}}$: RTvsHIIT+RT 0.61 ±0.22; RTvsRT+HIIT 0.56 ±0.23; ${\dot{\text{W}}}_{\text{LT}}$: RTvsHIIT+RT 0.87 ±0.47; RTvsRT+HIIT 0.74 ±0.51; ${\dot{\text{W}}}_{\text{peak}}$: RTvsHIIT+RT 0.68 ±0.29; RTvsRT+HIIT 0.65 ±0.29). There were no clear differences between concurrent exercise orders for any variable, at any time point (Fig 4c–4e and Table 2). Comparing the SDs of the changes PRE-to-POST with the SD of the changes in the reliability study (S1 Appendix), individual responses were evident in some groups for absolute ${\dot{\text{V}}\text{O}}_{2\text{peak}}$ (RT 5.9% vs 4.4%), ${\dot{\text{W}}}_{\text{LT}}$ (RT 17.1%, RT+HIIT 12.8% vs 5.5%), and ${\dot{\text{W}}}_{\text{peak}}$ (RT 6.7%, HIIT+RT 7.1%, RT+HIIT 7.5%, vs 4.5%).

### Training load

All groups completed similar *external* loads in the endurance and resistance sessions, respectively (Table 3). The HIIT+RT group perceived a greater resistance exercise internal load than both RT (*mean diff ± 90%CI*: 25 ± 20%, *ES ±90% CI*: 0.84 ±0.60) and RT+HIIT (-18 ± 9.3%, -0.76 ±0.44); there was no clear difference between RT and RT+HIIT. There was also no difference between HIIT+RT and RT+HIIT for endurance exercise internal load.

**Table 3:** Average weekly training loads (both internal and external), and average “readiness-to-train” (RTT) scores for the prescribed training sessions (mean ± SD).

	RT *(n = 9)*	HIIT+RT *(n = 10)*	RT+HIIT *(n = 10)*
**Training compliance (%)**	98 ± 3	96 ± 4	95 ± 5
**Resistance Sessions**			
Average Internal Load (AU)	269 ± 100	337 ± 84 ^A^^,^^C^	275 ± 76
Absolute Volume Load (kg)*	14,800 ± 3,900	14,200 ± 4,500	14,600 ± 3,400
RTT total score	18.1 ± 2.7	16.3 ± 2.9 ^A^	17.5 ± 3.4
*Fatigue*	3.3 ± 0.8	3.0 ± 0.8	3.2 ± 1.0
*Sleep*	3.6 ± 0.8	3.4 ± 0.8	3.5 ± 0.9
*General Muscle Soreness*	3.4 ± 0.9	2.9 ± 0.8 ^A^	3.1 ± 1.0
*Stress*	3.8 ± 0.6	3.4 ± 0.8 ^A^	3.7 ± 0.7
*Mood*	4.0 ± 0.5	3.6 ± 0.7 ^A^^,^^C^	4.0 ± 0.6
**Endurance Sessions**			
Average Internal Load	-	218 ± 71	217 ± 49
Absolute Work Done (kJ)	-	997 ± 230	929 ± 190
RTT total score	-	17.1 ± 3.1	17.3 ± 3.3
*Fatigue*	-	3.2 ± 0.9	3.2 ± 0.9
*Sleep*	-	3.4 ± 0.9	3.5 ± 0.9
*General Muscle Soreness*	-	3.3 ± 0.9	3.0 ± 0.9
*Stress*	-	3.5 ± 0.8	3.7 ± 0.7
*Mood*	-	3.7 ± 0.7	3.9 ± 0.6

^A^ = difference vs RT;

^C^ = difference vs RT+HIIT. Capital letters denote effects remained clear after adjustment for multiple comparisons.

*sets × reps × weight lifted (kg)

### Readiness-to-train

Before the resistance sessions, the HIIT+RT group reported lower (i.e., worse) scores than RT for total wellbeing (*raw mean difference ± 90% CI*: -1.8 ± 1.4, *ES ±90% CI*: -0.60 ±0.46), general muscle soreness (-0.6 ± 0.4, -0.62 ±0.46), stress (-0.4 ± 0.3, -0.51 ±0.40), and mood (-0.4 ± 0.2, -0.68 ±0.39). Mood scores were also greater in RT+HIIT than HIIT+RT (0.4 ± 0.3, 0.65 ±0.47). There were no differences between RT and RT+HIIT for any measure. Before the endurance sessions, there were no clear differences between HIIT+RT and RT+HIIT for any measure (Table 3).

## Discussion

We discovered in healthy, moderately-active men that concurrent training improved both maximal strength and lean mass comparably to resistance-only training, irrespective of the exercise order. Furthermore, both concurrent groups (HIIT+RT and RT+HIIT), but not RT, similarly improved ${\dot{\text{V}}\text{O}}_{2\text{peak}}$, ${\dot{\text{W}}}_{\text{LT}}$, and ${\dot{\text{W}}}_{\text{peak}}$, independent of exercise order. However, compared to RT, performing concurrent RT+HIIT training for nine weeks attenuated the development of CMJ displacement, force, and power. Only RT+HIIT training induced a meaningful reduction in total fat mass.

### Countermovement jump performance

Nine weeks of resistance-only training improved most measures of CMJ performance (with the exception of force expressed relative to body mass). In comparison, RT+HIIT mitigated the development of CMJ displacement, as well as absolute force and power. Our findings are congruent with previous reports of attenuated CMJ force and power [35, 63], and jump height [39, 63, 64] following concurrent training. We are uncertain why CMJ force and power were reduced following RT+HIIT; however, since strength and total lean mass were unaffected, we speculate that other neuromuscular mechanisms may be responsible. Previous studies have demonstrated reductions in voluntary rapid activation of trained muscles [38] with concurrent versus single-mode training, subsequently limiting the force-generating capacity of the muscle. However, as we did not measure changes in voluntary activation, it is difficult to provide a mechanistic explanation for the divergent between-group changes in the CMJ parameters. Nonetheless, our findings lend support to the notion that force and power production may be more susceptible to the interference effect than muscle strength and hypertrophy [10, 65], particularly when resistance exercise is performed first. Of note, similar attenuations were not evident in the HIIT+RT group. It is possible that the whole-body RT session may have induced greater, and longer-lasting neuromuscular fatigue than that of the HIIT cycling session, which when performed after RT, compromised neuromuscular adaptations governing force-generating capacity. Clearly, more research is needed to determine the neuromuscular mechanisms by which HIIT interferes with RT-induced improvements in CMJ performance. Nonetheless, the present results suggest that if the aim is to maximise power-related adaptations, where possible, RT should be performed in isolation (i.e., ≥ 24 h recovery between sessions), or if performed on the same day, scheduled at least 3 hours after HIIT.

### Maximal strength

Endurance exercise has been proposed to ‘acutely’ hinder resistance training quality, either through exercise-induced residual fatigue, or an anticipatory reduction in effort (if resistance training is performed first) [12, 36, 66]. In the present study, resistance training volume and subsequent improvements in 1-RM strength were similar following both resistance-only and concurrent training (irrespective of exercise order), corroborating previous findings [4, 67–72]. Furthermore, the observed improvement (~24–28%) is typical of 1-RM leg press gains previously-reported in similar populations [73–75].

We previously demonstrated attenuated strength gains following 8 weeks of concurrent HIIT+RT training, when sessions were performed in close proximity (10 min) [35]. In the present study, we adopted a more ecologically-valid between-mode recovery period of 3 hours, as is often utilised in many athletic populations [40–43]. This duration may have afforded participants sufficient time to recover, and to implement nutritional strategies to support subsequent exercise performance and training adaptations. Given the matched *external* resistance volume-loads across all three groups, it appears the performance of the second session, and thus the stimulus for strength development, was unimpeded by the prior HIIT session, thereby preventing an interference effect. However, the “readiness-to-train” and *internal* load data suggest performing HIIT first reduced perceptions of readiness prior to, and increased perceptions of effort during, the resistance training sessions compared to RT and RT+HIIT, who performed resistance exercise first. The physiological stress associated with a prior bout of HIIT may have induced some residual fatigue or muscle soreness that had not fully dissipated during the 3-hour recovery, whilst the RT group had a lower *total* training volume and longer recovery period between training sessions, possibly contributing to lower perceptions of soreness. While this did not translate into impaired resistance training loads and strength gains, it remains speculative whether a longer training program duration would have elicited more observable differences in both internal and external loads, and subsequent strength adaptations. Indeed, others have observed greater increases in training loads over 12 weeks when performing resistance training sessions first, compared to the reverse order [19], and attenuated strength gains only after 8 weeks of training [76, 77]. Despite influencing subjective measures, it is possible the HIIT modality (cycling) and intensity did not impair neuromuscular function to a degree necessary to reduce subsequent RT performance and volume-loads. However, it is also worth noting that the “readiness-to-train” questionnaire was conducted before the warm-up, and perceptions of readiness may have improved afterwards, as has been observed elsewhere [78]. Nonetheless, the present short-term concurrent training program, regardless of exercise order, did not limit the development of maximal, lower-body dynamic strength, compared to resistance-only training, in healthy, moderately-active men.

### Body composition

Although changes in total lean mass were only considered meaningful for RT+HIIT (both RT and HIIT+RT induced “*possible”* changes), all groups showed trivial-to-small positive effects, the differences between which were trivial. We have previously demonstrated attenuations to DXA-derived increases in lower-body lean mass, when resistance training was performed 10 minutes after HIIT cycling [35]. However, much of the existing literature does not support an interference effect of endurance exercise on hypertrophy [9], and attenuations in strength have been observed without compromised hypertrophy [76]. A recent meta-analysis also reported no interfering effect of concurrent HIIT and RT on both total and lower-body lean mass [79]. High-intensity contractions recruit more high-threshold, fast-twitch motoneurons [80], and the biomechanical similarities between the HIIT cycling and lower-body resistance exercises [81] may have provided a synergistic, rather than antagonistic, hypertrophic stimulus to a greater range of muscle fibres [9, 79]. Finally, consistent with recommendations for maximising post-exercise protein synthesis [58], participants were provided with protein after every session (0.25 g^.^kg_BM_^-1^ [≈ 20 g protein, ≈ 2 g leucine/serving]). They were also habitually consuming ~1.9-2.1 g^.^kg_BM_^.^d^-1^ of protein (determined via food diaries conducted at baseline), which is in excess of the upper limit (~1.6 g^.^kg_BM_^.^d^-1^) for maximising resistance training-induced changes in fat-free mass [82].

Concurrent RT+HIIT training reduced total fat mass, whilst both HIIT+RT and RT induced only trivial changes. Similar reductions (-16.2%) have been observed in sedentary men undergoing 8 weeks of performing resistance training 15–20 minutes before running-based endurance sessions [83]. A prior bout of resistance exercise has been shown to increase oxygen uptake [84, 85] and fat metabolism [86, 87] during subsequent endurance exercise. As such, performing resistance exercise before endurance exercise may promote greater overall energy expenditure and potentially fat metabolism. However, others have demonstrated no order effect on oxygen consumption both during [88, 89] and after concurrent sessions [90]. Rather than the training program *per se*, it is also possible the divergent group changes in fat mass may relate to changes in overall energy intake over the course of the training program. A limitation of the study is that habitual energy intake was only quantified using food diaries at baseline. However, using the body composition data and assumed energy densities of fat-free mass (1 kcal^.^g^-1^) and fat mass (9.5 kcal^.^g^-1^) [91–93], we were able to estimate changes in energy balance between time-points, which were almost perfectly correlated with changes in fat mass (*PRE-to-MID r* = 0.980, R^2^ = 0.960; *PRE-to-POST r* = 0.987, R^2^ = 0.974). Furthermore, the majority of RT+HIIT participants (depicted in S2 Fig) appear to have been in a negative energy balance throughout the study, whilst the HIIT+RT and RT cohorts appear more evenly split across positive and negative energy states. Collectively, the results suggest that greater fat mass losses are achieved when under a greater energy deficit, regardless of the different training modes completed herein.

### Aerobic fitness

Unlike resistance training adaptations, the development of aerobic capacity may not be as susceptible to the interference effect [10], though some evidence indicates acute endurance performance may still be compromised by prior resistance exercise-induced muscle soreness and damage, neuromuscular fatigue, reduced movement efficiency, and muscle glycogen depletion [94]. Thus, training in such a way may diminish subsequent endurance-related adaptations. Indeed, performing HIIT *before* RT for 12 weeks improved ${\dot{\text{V}}\text{O}}_{2\text{max}}$ and 4-km time trial performance more than the reverse order [23]. Others demonstrated greater improvements in ${\dot{\text{V}}\text{O}}_{2\text{peak}}$ when RT and HIIT sessions were separated by 24 h, compared to 0 h and 6 h [43]. Our concurrent training program, with only 3 h between sessions, induced small-to-moderate improvements in ${\dot{\text{V}}\text{O}}_{2\text{peak}}$, ${\dot{\text{W}}}_{\text{LT}}$, and ${\dot{\text{W}}}_{\text{peak}}$ regardless of exercise order, whilst RT training did not. The conflicting findings to those demonstrating order-dependent [23] and sub-optimal [43] endurance adaptations are likely due to the different endurance modalities employed. The previous studies utilised running [23, 43], whereas we employed cycling. Performing resistance exercise first has been shown to impair subsequent running performance more than the reverse order [13]. Furthermore, running is weight-bearing, and may induce greater physiological stress than cycling due to the greater eccentric loading involved [95]. It is possible that residual neuromuscular fatigue from the prior RT sessions may have compromised running performance (and thus, the stimulus for endurance-related adaptations) to a greater extent in the previous studies, and necessitated a longer recovery between modes than if combined with HIIT cycling [95], which also bares greater biomechanical similarities to the RT exercises performed than running [81]. The present findings could be expected, given the similar internal and external endurance training loads in both concurrent groups. Furthermore, the improvements in relative ${\dot{\text{V}}\text{O}}_{2\text{peak}}$ (~8 to 9%) are commensurate with other studies using similar participant cohorts, showing neither an interference [8, 37, 76, 96], nor an exercise order effect [20, 22, 27, 30, 32] on endurance capacity. Whilst the mechanisms contributing to the improved aerobic fitness in the present study were not determined, both concurrent groups displayed robust increases in aerobic capacity and power, supporting the view that the development of aerobic fitness is not limited by the addition of resistance training. However, we did not include any direct measures of indices of endurance performance (e.g., fixed distance or work-based time trial performance, tests for economy of movement), which may be affected by concurrent training session order and recovery duration (see [94, 95]).

### Other considerations

Although this study specifically investigated the role of exercise order, the effects of other training and “*non-training*” variables cannot be ignored when interpreting the results [97]. For example, a training frequency > 3 d^.^wk^-1^ may increase the likelihood of an interference effect [10, 76, 98]. Furthermore, prolonging the between-mode recovery duration (> 8-24 hours) may further benefit resistance exercise performance [16], strength [6, 36], hypertrophy [6, 7], and aerobic adaptations [22]. The relatively short (9-week) training period and the participants’ training status may also have affected the observed adaptations. When unaccustomed to a particular training regimen, several generic adaptations are induced, independent of the type of stimulus [99–102], whereas an interference effect, particularly to strength, may become more apparent in highly-trained individuals with prolonged training backgrounds (and for whom the potential for adaptation is comparatively lower) [103].

We also acknowledge the small sample sizes of each group, which we have determined would need to be 1.1-2.6× greater for the unclear mean effects to have acceptably narrow compatibility intervals. Furthermore, many of the standard deviations of the within-group change scores exceeded the SDs of the change scores derived in the reliability study (S1 Appendix), by a sufficient margin to indicate reasonable evidence for individual responses in all groups. The individual responses also appeared to be greater in some groups than in others; however, the small sample size precluded between-group comparisons of the individual responses [104].

## Conclusions

Concurrent exercise order does not appear to mediate the interference to hallmark resistance or endurance adaptations in moderately-active men, over a short-term training period. However, some measures of countermovement jump performance (peak displacement, force, and power) were compromised, particularly when RT was performed before HIIT. Furthermore, the exercise order may alter the participants’ motivation to train, by negatively affecting their subjective perceptions of effort during resistance exercise sessions when performed after endurance exercise sessions (i.e., HIIT+RT order). Whilst the long-term implications (months-to-years) of training in this way cannot be determined from the present work, healthy, moderately-active individuals engaging in same-day concurrent training with short recovery durations may wish to choose an exercise order based on individual preference, or, perhaps more importantly, periodised according to the most important goals of a specific training phase.

## Supporting information

S1 Appendix Reliability data for each primary variable. (PDF)

S2 Appendix Training program details. (PDF)

S3 Appendix Subjective questionnaires and scales, and 1-RM prediction table. (PDF)

S4 Appendix Summary of all within-group and between-group comparisons. (PDF)

S1 Fig Percent changes in (A) upper- and (B) lower-body lean mass after 5 (MID) and 9 (POST) weeks of training. (TIF)

S2 Fig Correlations between changes in fat mass and estimated energy balance, from (A) PRE-to-MID, and (B) PRE-to-POST training. (TIF)

## Abbreviations

${\dot{\text{V}}\text{O}}_{2\text{peak}}$: peak rate of oxygen uptake
${\dot{\text{W}}}_{\text{LT}}$: power at the lactate threshold
${\dot{\text{W}}}_{\text{peak}}$: peak aerobic power
1-RM-1: repetition maximum
AU: arbitrary units
BASE: baseline trial
CMJ: countermovement jump
d^.^wk^-1^: days per week
DXA: dual-energy x-ray absorptiometry
ES: standardised effect size
FAM: familiarisation trial
g^.^kg_BM_^-1^: grams per kilogram of body mass
GXT: graded exercise test
HIIT+RT: high-intensity interval training before resistance training group
HIIT: high-intensity interval training
kcal^.^kg_BM_^.^d^-1^: kilocalorie per kilogram per day
kJ: kilojoule
L^.^min^-1^: litres per minute
m^.^s^-1^: metres per second
MID: mid-training trial
mL^.^kg^.^min^-1^: millilitres per kilogram of body mass per minute
N^.^kg_BM_^-1^: newtons per kilogram of body mass
N: newton
POST: post-training trial
RIR: repetitions in reserve
RM: repetition maximum
RPE: rating of perceived exertion
RPM: revolutions per minute
RT+HIIT: resistance before high-intensity interval training group
RT: resistance-only training group
SD: standard deviation
sRPE: session rating of perceived exertion
VB: verification bout
W^.^kg_BM_^-1^: watts per kilogram of body mass
W: watt
