# Supplementary material for: Order of same-day concurrent training influences some indices of power development, but not strength, lean mass, or aerobic fitness in healthy, moderately-active men after 9 weeks of training
Source: PLoS One. 2020 May 14;15(5):e0233134. doi: 10.1371/journal.pone.0233134 (PMC7224562; doi:10.1371/journal.pone.0233134)
Supplement: S1 Appendix — (PDF) [file pone.0233134.s001.pdf]

Appendix S1

The technical error of measurement (TEM), the coefficient of variation (CV), and the intra-class correlation coefficient were calculated for all primary variables, using data from *FAM2* and the *BASE* trials (Table SM1). The reliability data were determined using Excel spreadsheets specifically formulated for the analysis of reliability [1]. It should be noted that the data in Table 3.2 represent the reliability of these measures when the repeated tests were separated by 48 hours to 1 week. Given that these tests were used to assess changes in performance measures before and after 9 weeks of training, it would have been more appropriate to assess the reliability of the tests when separated by a 10-week control period; however, due to the time constraints of this project, this was not possible.

Table SM1 - Technical error of measurement (TEM), the coefficient of variation (CV) and the intra-class correlation coefficient (ICC) for each primary variable, with lower and upper 90% compatibility limits

	**TEM**	**CV (%)**	***SD (%)***	**ICC**
Lower-Body Maximal Strength				
Leg press 1-RM (kg)	10.0 (*8.3 – 12.9*)	3.5 (*2.8 – 4.5*)	*4.9*	0.99 (*0.98 – 0.99*)
Countermovement Jump Variables				
Peak Displacement (m)	0.02 (*0.01 – 0.02*)	4.4 (3.6 – *5.8*)	*6.2*	0.93 (*0.88 – 0.96*)
Peak Velocity (m/s)	0.06 (*0.05 – 0.07*)	2.1 (*1.7 – 2.7*)	*3.0*	0.92 (*0.85 – 0.96*)
Peak Force (N)	66 (*54 – 85*)	6.7 (5.5 – *8.7*)	*9.5*	0.92 (*0.85 – 0.95*)
Peak Power (W)	132 (*109 – 170*)	3.5 (*2.9 – 4.6*)	*4.9*	0.97 (*0.94 – 0.98*)
Body Composition				
Total LBM (kg)	0.44 (*0.36 – 0.58*)	0.9 (*0.7 – 1.1*)	*1.3*	0.997 (*0.994 – 0.998*)
Upper LBM (kg)	0.48 (*0.39 – 0.62*)	1.5 (*1.2 – 1.9*)	*2.1*	0.990 (*0.981 – 0.995*)
Lower LBM (kg)	0.20 (*0.16 – 0.26*)	1.1 (*0.9 – 1.5*)	*1.6*	0.996 (*0.992 – 0.998*)
Total Fat Mass (kg)	0.37 (*0.31 – 0.49*)	2.9 (*2.4 – 3.8*)	*4.1*	0.997 (*0.993 – 0.998*)
Aerobic Fitness				
Absolute $\dot{V}O_{2peak}$ (L/min)	0.11 (*0.09 – 0.14*)	3.1 (*2.6 – 4.1*)	*4.4*	0.97 (*0.95 – 0.99*)
Relative $\dot{V}O_{2peak}$ (mL/kg/min)	1.4 (*1.2 – 1.9)*	3.1 (*2.5 – 4.0*)	*4.4*	0.96 (*0.93 – 0.98*)
Lactate Threshold, W_{LT} (W)	6 (*5 – 8*)	3.9 (*3.2 – 5.1*)	*5.5*	0.97 (*0.95 – 0.99*)
Peak Aerobic Power, W_{peak} (W)	7 (*6 – 9*)	3.2 (2.6 – *4.1*)	*4.5*	0.98 (*0.96 – 0.99*)

SD standard deviation, calculated as [$\sqrt{2}$×CV]

Reference

1. Hopkins WG. Spreadsheets for analysis of validity and reliability. Sportscience. 2017;21.
