# Supplementary material for: Order of same-day concurrent training influences some indices of power development, but not strength, lean mass, or aerobic fitness in healthy, moderately-active men after 9 weeks of training
Source: PLoS One. 2020 May 14;15(5):e0233134. doi: 10.1371/journal.pone.0233134 (PMC7224562; doi:10.1371/journal.pone.0233134)
Supplement: S2 Appendix — (PDF) [file pone.0233134.s002.pdf]

Appendix S2

Training Program Details

Week 1

Week 1 differed slightly from the subsequent 8 weeks of training, as this week also formed part of an acute experimental trial, investigating the molecular responses to concurrent training (*currently unpublished*). Whilst the molecular data are outside the scope of this paper and not discussed here, this was illustrated to highlight the differences between Week 1 and the rest of the 9-week training program.

The participants trained on 3 non-consecutive days, as per the rest of the study. However, on day 1, the participants completed an "*experimental training day*" during which muscle biopsies were also sampled at various timepoints to characterise temporal changes to protein and gene expression following resistance-only and concurrent exercise sessions. The exercise protocols in week 1 involved:

> **Resistance exercise**: 6×10 leg press repetitions, at 70 % 1-RM, with 2 minutes between sets.
>
> **Endurance exercise**: 10×2-minute cycling bouts, at 40 % of the difference between the power at the lactate threshold ($\dot{W}_{LT}$) and peak aerobic power ($\dot{W}_{peak}$) (≈ 85 ± 1 %$\dot{W}_{peak}$).

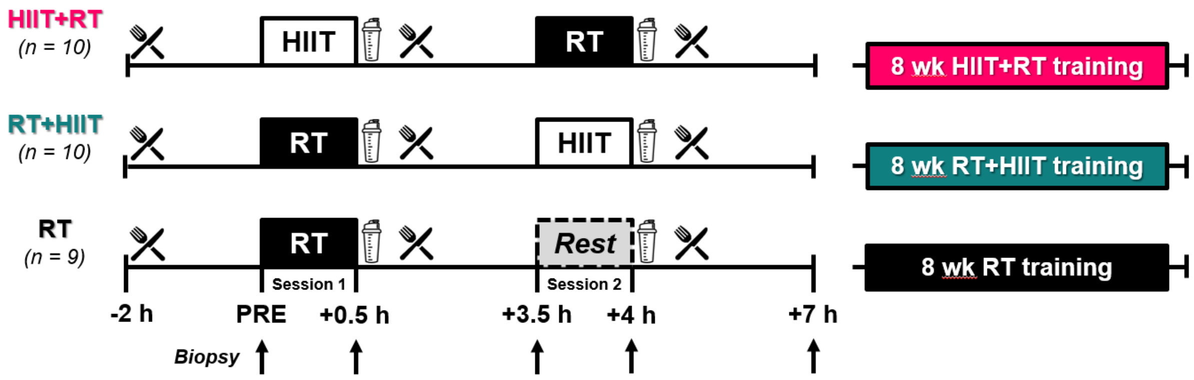

Figure S2.1 Schematic overview of the experimental training day in Week 1. *HIIT* high-intensity interval training; *RT resistance training only; HIIT+RT* high-intensity interval training and resistance training group; *RT+HIIT* resistance and high-intensity interval training group; ↑ = muscle biopsy; ✕ = standardised meal (carbohydrate = 1.3 $g \cdot kg_{BM}^{-1}$; protein = 0.3 $g \cdot kg_{BM}^{-1}$; fat = 0.3 $g \cdot kg_{BM}^{-1}$); = whey protein (0.25 $g \cdot kg_{BM}^{-1}$).

Weeks 2 to 9

Table S2.1 *8-week resistance training programme, adapted from [1]*

	Week:	2	3	4	5		6	7	8	9	Exercises:
MON and FRI	*Sets:*	3	3	3	3	MID Testing	4	4	4	4	*Leg press, bench press, seated row, leg extension, leg curl*
	RM:	12	10	8	6		12	10	8	6	
WED	*Sets:*	3	3	3	3		4	4	4	4	*Leg press, dumbbell chest press, lat. pulldown, lunges, leg curl*
	RM:	12	12	10	10		8	8	6	6	

RM = rep. max target

Table S2.2 *8-week endurance training programme, adapted from [1].*

Week . Session	No. of 2-min Intervals	Training Intensity ($\dot{W}_{Ex}$)†
2.1	8	χ = 50-60% (≈ 87 ± 1 %$\dot{W}_{peak}$)
2.2	9	
2.3	10	
3.1	9	χ = 55-65% (≈ 89 ± 1 %$\dot{W}_{peak}$)
3.2	11	
3.3	10	
4.1	11	χ = 60-70% (≈ 90 ± 1 %$\dot{W}_{peak}$)
4.2	12	
4.3	11	
5.1	10	χ = 65-75% (≈ 91 ± 1 %$\dot{W}_{peak}$)
5.2	9	
5.3	8	
*MID**	8	χ = 60% ‡ (≈ 91 ± 2 %$\dot{W}_{peak}$)
6.1	9	χ = 65-75% (≈ 93 ± 1 %$\dot{W}_{peak}$)
6.2	10	
6.3	11	
7.1	10	χ = 70-80% (≈ 94 ± 1 %$\dot{W}_{peak}$)
7.2	11	
7.3	12	
8.1	11	χ = 75-85% (≈ 95 ± 1 %$\dot{W}_{peak}$)
8.2	13	
8.3	12	
9.1	11	χ = 80-90% (≈ 97 ± 1 %$\dot{W}_{peak}$)
9.2	9	
9.3	8	
*POST**	8	χ = 80% ‡ (≈ 96 ± 1 %$\dot{W}_{peak}$)

† Training intensity calculated as: $\dot{W}_{LT} + (\chi\,\% \times [\dot{W}_{peak} - \dot{W}_{LT}])$.

** Additional training session added at the end of MID- and POST-testing weeks, respectively.*

‡ Training intensity re-calculated using updated data from MID- and POST-testing weeks, respectively.

Reference:

1. Fyfe JJ, Bartlett JD, Hanson ED, Stepto NK, Bishop DJ. Endurance training intensity does not mediate interference to maximal lower-body strength gain during short-term concurrent training. Frontiers in physiology. 2016;7:487. Epub 2016/11/20. doi: 10.3389/fphys.2016.00487. PubMed PMID: 27857692.
