# Supplementary material for: Order of same-day concurrent training influences some indices of power development, but not strength, lean mass, or aerobic fitness in healthy, moderately-active men after 9 weeks of training
Source: PLoS One. 2020 May 14;15(5):e0233134. doi: 10.1371/journal.pone.0233134 (PMC7224562; doi:10.1371/journal.pone.0233134)
Supplement: S3 Appendix — (PDF) [file pone.0233134.s003.pdf]

Appendix S3

Wellness/Readiness to train questionnaire

Reference: *[1]*

	5	4	3	2	1	TOTAL
**Fatigue**	Very fresh	Fresh	Normal	More tired than normal	Always tired	
**Sleep Quality**	Very restful	Good	Difficulty falling asleep	Restless sleep	Insomnia	
**General muscle soreness**	Feeling great	Feeling good	Normal	Increase in soreness/tightness	Very sore	
**Stress Levels**	Very relaxed	Relaxed	Normal	Feeling stressed	Highly stressed	
**Mood**	Very positive mood	A generally good mood	Less interested than usual in others and/or activities	Snappiness at teammates, family, and/or co-workers	Highly annoyed/ irritable/ down	

Appendix S3

Session rating of perceived exertion (sRPE)

Reference: *[2]*

Session Rate of Perceived Exertion (sRPE)

Rating = Descriptor

0 = Rest

1 = Very, very easy

2 = Easy

3 = Moderate

4 = Somewhat Hard

5 = Hard

6 = *

7 = Very Hard

8 = *

9 = *

10 = Maximal

Appendix S3

Repetitions in Reserve (RIR) Scale

Reference: *[3]*

Resistance exercise-specific RPE – Reps. in Reserve

Rating	Description of Perceived Exertion
**10**	*Maximum effort – no further reps*
**9.5**	*No further reps, but could increase load*
**9**	*1 rep remaining*
**8.5**	*1-2 reps remaining*
**8**	*2 reps remaining*
**7.5**	*2-3 reps remaining*
**7**	*3 reps remaining*
**5-6**	*4-6 reps remaining*
**3-4**	*Light effort*
**1-2**	*Little to no effort*

Appendix S3

1-RM Prediction Table

Reference: *[4]*

Max Reps (RM)	1	2	3	4	5	6	7	8	9	10	12	15
%1RM	100	95	93	90	87	85	83	80	77	75	67	65
Load (lbs or kg)	10	10	9	9	9	9	8	8	8	8	7	7
	20	19	19	18	17	17	17	16	15	15	13	13
	30	29	28	27	26	26	25	24	23	23	20	20
	40	38	37	36	35	34	33	32	31	30	27	26
	50	48	47	45	44	43	42	40	39	38	34	33
	60	57	56	54	52	51	50	48	46	45	40	39
	70	67	65	63	61	60	58	56	54	53	47	46
	80	76	74	72	70	68	66	64	62	60	54	52
	90	86	84	81	78	77	75	72	69	68	60	59
	100	95	93	90	87	85	83	80	77	75	67	65
	110	105	102	99	96	94	91	88	85	83	74	72
	120	114	112	108	104	102	100	96	92	90	80	78
	130	124	121	117	113	111	108	104	100	98	87	85
	140	133	130	126	122	119	116	112	108	105	94	91
	150	143	140	135	131	128	125	120	116	113	101	98
	160	152	149	144	139	136	133	128	123	120	107	104
	170	162	158	153	148	145	141	136	131	128	114	111
	180	171	167	162	157	153	150	144	139	135	121	117
	190	181	177	171	165	162	158	152	146	143	127	124
	200	190	186	180	174	170	166	160	154	150	134	130
	210	200	195	189	183	179	174	168	162	158	141	137
	220	209	205	198	192	187	183	176	170	165	148	143
	230	219	214	207	200	196	191	184	177	173	154	150
	240	228	223	216	209	204	199	192	185	180	161	156
	250	238	233	225	218	213	208	200	193	188	168	163
	260	247	242	234	226	221	216	208	200	195	174	169
	270	257	251	243	235	230	224	216	208	203	181	176
	280	266	260	252	244	238	233	224	216	210	188	182
	290	276	270	261	253	247	241	232	224	218	195	189
	300	285	279	270	261	255	249	240	231	225	201	195
	310	295	288	279	270	264	258	248	239	233	208	202
	320	304	298	288	279	272	266	256	247	240	215	208
	330	314	307	297	287	281	274	264	254	248	221	215
	340	323	316	306	296	289	282	272	262	255	228	221
	350	333	325	315	305	298	291	280	270	263	235	228
	360	342	335	324	313	306	299	288	277	270	241	234
	370	352	344	333	322	315	307	296	285	278	248	241
	380	361	353	342	331	323	316	304	293	285	255	247
	390	371	363	351	340	332	324	312	301	293	262	254
	400	380	372	360	348	340	332	320	308	300	268	260

Appendix S3

	410	390	381	369	357	349	341	328	316	308	275	267
	420	399	391	378	366	357	349	336	324	315	282	273
	430	409	400	387	374	366	357	344	331	323	288	280
	440	418	409	396	383	374	366	352	339	330	295	286
	450	428	418	405	392	383	374	360	347	338	302	293
	460	437	428	414	401	391	382	368	355	345	309	299
	470	447	437	423	409	400	390	376	362	353	315	306
	480	456	446	432	418	408	399	384	370	360	322	312
	490	466	456	441	427	417	407	392	378	368	329	319
	500	475	465	450	435	425	415	400	385	375	335	325
	510	485	474	459	444	434	424	408	393	383	342	332
	520	494	484	468	453	442	432	416	401	390	349	338
	530	504	493	477	461	451	440	424	408	398	355	345
	540	513	502	486	470	459	449	432	416	405	362	351
	550	523	511	495	479	468	457	440	424	413	369	358
	560	532	521	504	488	476	465	448	432	420	376	364
	570	542	530	513	496	485	474	456	439	428	382	371
	580	551	539	522	505	493	482	464	447	435	389	377
	590	561	549	531	514	502	490	472	455	443	396	384
	600	570	558	540	522	510	498	480	462	450	402	390

References:

1. McLean BD, Coutts AJ, Kelly V, McGuigan MR, Cormack SJ. Neuromuscular, endocrine, and perceptual fatigue responses during different length between-match microcycles in professional rugby league players. International journal of sports physiology and performance. 2010;5(3):367-83.

2. Foster C, Florhaug JA, Franklin J, Gottschall L, Hrovatin LA, Parker S et al. A new approach to monitoring exercise training. Journal of strength and conditioning research / National Strength & Conditioning Association. 2001;15(1):109-15.

3. Zourdos MC, Klemp A, Dolan C, Quiles JM, Schau KA, Jo E et al. Novel Resistance Training-Specific Rating of Perceived Exertion Scale Measuring Repetitions in Reserve. Journal of strength and conditioning research / National Strength & Conditioning Association. 2016;30(1):267-75. doi:10.1519/jsc.0000000000001049.

4. Baechle TR, Earle RW. Essentials of strength training and conditioning. Champaign, IL: Human kinetics; 2008.
