# Supplementary material for: Order of same-day concurrent training influences some indices of power development, but not strength, lean mass, or aerobic fitness in healthy, moderately-active men after 9 weeks of training
Source: PLoS One. 2020 May 14;15(5):e0233134. doi: 10.1371/journal.pone.0233134 (PMC7224562; doi:10.1371/journal.pone.0233134)
Supplement: S4 Appendix — (PDF) [file pone.0233134.s004.pdf]

PRE-to-MID within-group and between-group changes in performance variables

For within-group changes (PRE vs MID), and the differences in changes between groups (RT vs HIIT+RT and RT vs RT+HIIT):

p^{H} value threshold for rejection of harm: < 0.005

p^{B} value threshold for rejection of benefit: < 0.25

For the differences in changes between HIIT+RT vs RT+HIIT:

$p^{-/+}$ value threshold for rejection of substantially positive (+ve) or negative (-ve) effect: < 0.05

%Δ = mean percent change; %diff = mean percent difference in change scores; SD = standard deviation; ES = standardised effect size (Cohen's d); 90%CI = 90% compatibility interval;
↑= substantially beneficial or positive effect (i.e., improved); ↔ = trivial effect; ↓ = substantially harmful or negative effect (i.e., impaired)
@25/.5% = 25% chance of benefit, 0.5% risk of harm; @5/.1% = 5% chance of beneit, 0.1% risk of harm
@90% = 90% compatibility interval; @99% = 99% compatibility interval
Bold text indicates effects which remained clear at more conservative thresholds after adjusting for multiple inferences.
TBLH = Total Body Less Head

Within-Group Changes (Δ)		Percent Effect %Δ ± SD	Standardised Effect Size (ES) ES (d) ± 90% CL	Magnitude	Likelihood true effect is ↑/↔/↓	Threshold for clear effect:	p^{H} value (harm)	p^{B} value (benefit)	p value (NHST)
**Leg Press 1-RM**	**RT**	15.1 ± 4.6	0.49 ± 0.14	Small	**Most Likely ↑**	**@5/.1%**	< 0.0005	0.999	< 0.0005
	**HIIT+RT**	17.7 ± 7.0	0.56 ± 0.18	Small	**Most Likely ↑**	**@5/.1%**	< 0.0005	0.999	< 0.0005
	**RT+HIIT**	16.9 ± 3.1	0.54 ± 0.14	Small	**Most Likely ↑**	**@5/.1%**	< 0.0005	1.000	< 0.0005
**Leg Press 1-RM (relative)**	**RT**	12.9 ± 3.5	0.57 ± 0.16	Small	**Most Likely ↑**	**@5/.1%**	< 0.0005	1.000	< 0.0005
	**HIIT+RT**	16.0 ± 6.8	0.70 ± 0.23	Moderate	**Most Likely ↑**	**@5/.1%**	< 0.0005	0.999	< 0.0005
	**RT+HIIT**	16.4 ± 2.7	0.71 ± 0.18	Moderate	**Most Likely ↑**	**@5/.1%**	< 0.0005	1.000	< 0.0005
**CMJ Height**	**RT**	6.9 ± 9.8	0.41 ± 0.40	Small		unclear	0.013	0.834	0.089
	**HIIT+RT**	2.4 ± 4.8	0.15 ± 0.17	Trivial	Possibly ↑	@25/.5%	0.003	0.296	0.150
	**RT+HIIT**	-2.2 ± 4.6	-0.14 ± 0.16	Trivial	**Possibly ↓**	**@5/.1%**	0.249	0.002	0.157
**CMJ Velocity**	**RT**	3.0 ± 4.2	0.42 ± 0.40	Small		unclear	0.013	0.840	0.087
	**HIIT+RT**	1.3 ± 2.1	0.18 ± 0.17	Trivial	Possibly ↑	@25/.5%	0.001	0.425	0.081
	**RT+HIIT**	-1.0 ± 2.0	-0.15 ± 0.17	Trivial	**Possibly ↓**	**@5/.1%**	0.295	0.002	0.135
**CMJ Force (absolute)**	**RT**	8.7 ± 9.6	0.37 ± 0.28	Small	Likely ↑	@25/.5%	0.002	0.864	0.035
	**HIIT+RT**	0.1 ± 11.2	0.00 ± 0.28	Trivial	Likely ↔	@25/.5%	0.109	0.115	0.985
	**RT+HIIT**	0.6 ± 11.6	0.03 ± 0.29	Trivial	Likely ↔	@25/.5%	0.091	0.150	0.866
**CMJ Force (relative)**	**RT**	5.8 ± 9.2	0.30 ± 0.32	Small		unclear	0.009	0.720	0.108
	**HIIT+RT**	0.1 ± 10.8	0.00 ± 0.33	Trivial	Possibly ↔	@25/.5%	0.141	0.149	0.986
	**RT+HIIT**	-0.7 ± 12.1	-0.04 ± 0.36	Trivial	Possibly ↔	@25/.5%	0.214	0.130	0.855
**CMJ Power (absolute)**	**RT**	9.7 ± 5.5	0.50 ± 0.21	Small	**Very Likely ↑**	**@5/.1%**	0.000	0.988	0.001
	**HIIT+RT**	1.9 ± 3.7	0.10 ± 0.12	Trivial	**Likely ↔**	**@5/.1%**	0.001	0.075	0.142
	**RT+HIIT**	-0.6 ± 4.2	-0.03 ± 0.13	Trivial	**Very Likely ↔**	**@5/.1%**	0.022	0.005	0.632
**CMJ Power (relative)**	**RT**	6.5 ± 5.7	0.42 ± 0.25	Small	**Likely ↑**	**@5/.1%**	< 0.0005	0.934	0.011
	**HIIT+RT**	1.1 ± 4.6	0.07 ± 0.18	Trivial	Likely ↔	@25/.5%	0.011	0.112	0.474
	**RT+HIIT**	-1.7 ± 5.5	-0.11 ± 0.21	Trivial	**Possibly ↔**	**@5/.1%**	0.239	0.012	0.348
**Total Lean Mass (TBLH)**	**RT**	2.0 ± 2.3	0.14 ± 0.10	Trivial	**Likely ↔**	**@5/.1%**	< 0.0005	0.153	0.035
	**HIIT+RT**	2.1 ± 1.3	0.15 ± 0.06	Trivial	**Likely ↔**	**@5/.1%**	< 0.0005	0.072	0.001
	**RT+HIIT**	2.5 ± 2.2	0.17 ± 0.09	Trivial	**Possibly ↑**	**@5/.1%**	< 0.0005	0.290	0.006
**Upper Lean Mass (TBLH)**	**RT**	1.4 ± 2.1	0.10 ± 0.09	Trivial	**Very Likely ↔**	**@5/.1%**	< 0.0005	0.042	0.080
	**HIIT+RT**	2.1 ± 1.3	0.15 ± 0.06	Trivial	**Likely ↔**	**@5/.1%**	< 0.0005	0.093	0.001
	**RT+HIIT**	2.1 ± 2.1	0.15 ± 0.09	Trivial	**Likely ↔**	**@5/.1%**	< 0.0005	0.159	0.016
**Lower Lean Mass (TBLH)**	**RT**	2.9 ± 3.7	0.18 ± 0.15	Trivial	**Possibly ↑**	**@5/.1%**	0.001	0.394	0.050
	**HIIT+RT**	2.3 ± 2.0	0.14 ± 0.08	Trivial	**Likely ↔**	**@5/.1%**	< 0.0005	0.108	0.008
	**RT+HIIT**	3.2 ± 3.6	0.20 ± 0.13	Small	**Possibly ↑**	**@5/.1%**	< 0.0005	0.503	0.020
**Total Fat Mass (TBLH)**	**RT**	2.4 ± 4.3	0.05 ± 0.06	Trivial	**Most Likely ↔**	**@5/.1%**	0.001	< 0.0005	0.138
	**HIIT+RT**	-1.6 ± 8.8	-0.04 ± 0.12	Trivial	**Very Likely ↔**	**@5/.1%**	0.002	0.015	0.569
	**RT+HIIT**	-6.7 ± 7.9	-0.16 ± 0.11	Trivial	**Possibly ↑**	**@5/.1%**	< 0.0005	0.264	0.019
**Absolute VO2peak**	**RT**	0.8 ± 4.5	0.04 ± 0.14	Trivial	**Very Likely ↔**	**@5/.1%**	0.007	0.038	0.597
	**HIIT+RT**	6.7 ± 5.0	0.33 ± 0.17	Small	**Likely ↑**	**@5/.1%**	< 0.0005	0.903	0.006
	**RT+HIIT**	6.3 ± 6.7	0.32 ± 0.22	Small	**Likely ↑**	**@5/.1%**	0.001	0.822	0.023
**Relative VO2peak**	**RT**	-2.3 ± 4.5	-0.14 ± 0.16	Trivial	**Likely ↔**	**@5/.1%**	0.244	0.003	0.158
	**HIIT+RT**	5.4 ± 4.8	0.31 ± 0.18	Small	**Likely ↑**	**@5/.1%**	< 0.0005	0.845	0.011
	**RT+HIIT**	5.5 ± 7.0	0.31 ± 0.25	Small	Likely ↑	@25/.5%	0.002	0.784	0.045
**Lactate Threshold**	**RT**	-0.2 ± 12.1	-0.01 ± 0.32	Trivial	Possibly ↔	@25/.5%	0.145	0.131	0.971
	**HIIT+RT**	15.0 ± 4.7	0.60 ± 0.17	Moderate	**Most Likely ↑**	**@5/.1%**	< 0.0005	1.000	< 0.0005
	**RT+HIIT**	9.7 ± 7.9	0.40 ± 0.21	Small	**Likely ↑**	**@5/.1%**	< 0.0005	0.943	0.005
**Peak Power**	**RT**	-1.9 ± 6.7	-0.09 ± 0.19	Trivial	**Likely ↔**	**@5/.1%**	0.158	0.012	0.401
	**HIIT+RT**	8.8 ± 7.6	0.39 ± 0.21	Small	**Likely ↑**	**@5/.1%**	< 0.0005	0.935	0.006
	**RT+HIIT**	7.7 ± 5.0	0.35 ± 0.15	Small	**Likely ↑**	**@5/.1%**	< 0.0005	0.945	0.001

Between-Group Differences in Δ		Percent Effect %diff ± 90%CL	Standardised Effect Size (ES) ES (d) ± 90% CL	Magnitude	Likelihood true effect is ↑/↔/↓	Threshold for clear effect:	$p^{H/-}$ value (harm/-ve)	$p^{B/+}$ value (bene/+ve)	p value (NHST)
**Leg Press 1-RM**	**RT vs HIIT+RT**	2.2 ± 4.7	0.08 ± 0.16	Trivial	Likely ↔	@25/.5%	0.004	0.100	0.411
	**RT vs RT+HIIT**	1.6 ± 3.3	0.06 ± 0.11	Trivial	**Very Likely ↔**	**@5/.1%**	0.001	0.020	0.391
	**HIIT+RT vs RT+HIIT**	-0.6 ± 4.2	-0.02 ± 0.15	Trivial	Very Likely ↔	@90%	0.026	0.010	0.796
**Leg Press 1-RM (relative)**	**RT vs HIIT+RT**	2.8 ± 4.3	0.13 ± 0.20	Trivial		unclear	0.006	0.266	0.272
	**RT vs RT+HIIT**	3.1 ± 2.6	0.14 ± 0.12	Trivial	**Likely ↔**	**@5/.1%**	< 0.0005	0.221	0.054
	**HIIT+RT vs RT+HIIT**	0.4 ± 4.0	0.02 ± 0.19	Trivial	Likely ↔	@90%	0.032	0.055	0.877
**CMJ Height**	**RT vs HIIT+RT**	-4.2 ± 6.3	-0.27 ± 0.41	Small	**Possibly ↓**	**@5/.1%**	0.616	0.034	0.255
	**RT vs RT+HIIT**	-8.4 ± 6.0	-0.55 ± 0.42	Small	**Likely ↓**	**@5/.1%**	0.922	0.005	0.037
	**HIIT+RT vs RT+HIIT**	-4.4 ± 3.4	-0.28 ± 0.23	Small	**Possibly ↓**	**@99%**	0.733	0.001	0.040
**CMJ Velocity**	**RT vs HIIT+RT**	-1.7 ± 2.8	-0.24 ± 0.41	Small	**Possibly ↓**	**@5/.1%**	0.566	0.041	0.302
	**RT vs RT+HIIT**	-3.9 ± 2.8	-0.57 ± 0.42	Small	**Likely ↓**	**@5/.1%**	0.930	0.004	0.034
	**HIIT+RT vs RT+HIIT**	-2.3 ± 1.5	-0.33 ± 0.23	Small	**Likely ↓**	**@99%**	0.827	< 0.0005	0.020
**CMJ Force (absolute)**	**RT vs HIIT+RT**	-8.0 ± 7.4	-0.37 ± 0.37	Small	**Likely ↓**	**@5/.1%**	0.786	0.008	0.092
	**RT vs RT+HIIT**	-7.4 ± 7.6	-0.35 ± 0.37	Small	**Possibly ↓**	**@5/.1%**	0.749	0.011	0.118
	**HIIT+RT vs RT+HIIT**	0.5 ± 8.5	0.02 ± 0.38	Trivial		unclear	0.159	0.216	0.912
**CMJ Force (relative)**	**RT vs HIIT+RT**	-5.4 ± 7.4	-0.30 ± 0.43	Small	**Possibly ↓**	**@5/.1%**	0.656	0.029	0.233
	**RT vs RT+HIIT**	-6.1 ± 7.8	-0.34 ± 0.45	Small	**Possibly ↓**	**@5/.1%**	0.701	0.027	0.204
	**HIIT+RT vs RT+HIIT**	-0.7 ± 8.5	-0.04 ± 0.46	Trivial		unclear	0.277	0.188	0.881
**CMJ Power (absolute)**	**RT vs HIIT+RT**	-7.1 ± 3.5	-0.40 ± 0.22	Small	**Likely ↓**	**@5/.1%**	0.932	< 0.0005	0.005
	**RT vs RT+HIIT**	-9.4 ± 3.5	-0.53 ± 0.24	Small	**Very Likely ↓**	**@5/.1%**	0.987	< 0.0005	0.001
	**HIIT+RT vs RT+HIIT**	-2.5 ± 2.9	-0.13 ± 0.16	Trivial	**Possibly ↓**	**@99%**	0.251	0.001	0.166
**CMJ Power (relative)**	**RT vs HIIT+RT**	-5.1 ± 3.9	-0.35 ± 0.29	Small	**Likely ↓**	**@5/.1%**	0.815	0.002	0.043
	**RT vs RT+HIIT**	-7.7 ± 4.1	-0.54 ± 0.32	Small	**Very Likely ↓**	**@5/.1%**	0.960	< 0.0005	0.006
	**HIIT+RT vs RT+HIIT**	-2.7 ± 3.8	-0.19 ± 0.26	Trivial	Possibly ↓	@90%	0.466	0.010	0.231
**Total Lean Mass (TBLH)**	**RT vs HIIT+RT**	0.1 ± 1.6	0.01 ± 0.11	Trivial	Very Likely ↔	@25/.5%	0.003	0.004	0.904
	**RT vs RT+HIIT**	0.4 ± 1.8	0.03 ± 0.13	Trivial	Very Likely ↔	@25/.5%	0.003	0.016	0.667
	**HIIT+RT vs RT+HIIT**	0.3 ± 1.4	0.02 ± 0.10	Trivial	**Most Likely ↔**	**@99%**	0.001	0.003	0.676
**Upper Lean Mass (TBLH)**	**RT vs HIIT+RT**	0.7 ± 1.4	0.05 ± 0.10	Trivial	**Very Likely ↔**	**@5/.1%**	< 0.0005	0.012	0.406
	**RT vs RT+HIIT**	0.6 ± 1.7	0.05 ± 0.12	Trivial	Very Likely ↔	**@25/.5%**	0.001	0.020	0.514
	**HIIT+RT vs RT+HIIT**	-0.1 ± 1.4	0.00 ± 0.10	Trivial	**Most Likely ↔**	**@99%**	0.002	0.002	0.946
**Lower Lean Mass (TBLH)**	**RT vs HIIT+RT**	-0.6 ± 2.4	-0.04 ± 0.15	Trivial	**Very Likely ↔**	**@5/.1%**	0.040	0.009	0.687
	**RT vs RT+HIIT**	0.4 ± 2.9	0.02 ± 0.18	Trivial	Likely ↔	@25/.5%	0.024	0.053	0.829
	**HIIT+RT vs RT+HIIT**	0.9 ± 2.3	0.06 ± 0.14	Trivial	**Likely ↔**	**@99%**	0.003	0.050	0.481
**Total Fat Mass (TBLH)**	**RT vs HIIT+RT**	-3.9 ± 5.2	-0.09 ± 0.13	Trivial	**Likely ↔**	**@5/.1%**	0.001	0.076	0.217
	**RT vs RT+HIIT**	-8.9 ± 4.5	-0.22 ± 0.12	Small	**Possibly ↑**	**@5/.1%**	< 0.0005	0.586	0.005
	**HIIT+RT vs RT+HIIT**	-5.2 ± 6.0	-0.12 ± 0.15	Trivial	**Likely ↔**	**@99%**	0.001	0.193	0.157
**Absolute VO2peak**	**RT vs HIIT+RT**	5.8 ± 4.1	0.29 ± 0.21	Small	**Likely ↑**	**@5/.1%**	< 0.0005	0.771	0.024
	**RT vs RT+HIIT**	5.4 ± 4.9	0.27 ± 0.25	Small	Possibly ↑	@25/.5%	0.002	0.697	0.064
	**HIIT+RT vs RT+HIIT**	-0.4 ± 4.8	-0.02 ± 0.25	Trivial		unclear	0.110	0.073	0.899
**Relative VO2peak**	**RT vs HIIT+RT**	7.9 ± 4.1	0.44 ± 0.24	Small	**Very Likely ↑**	**@5/.1%**	< 0.0005	0.952	0.003
	**RT vs RT+HIIT**	8.0 ± 5.2	0.45 ± 0.29	Small	**Likely ↑**	**@5/.1%**	0.001	0.921	0.013
	**HIIT+RT vs RT+HIIT**	0.1 ± 4.9	0.00 ± 0.29	Trivial		unclear	0.114	0.125	0.977
**Lactate Threshold**	**RT vs HIIT+RT**	15.2 ± 8.9	0.61 ± 0.35	Moderate	**Very Likely ↑**	**@5/.1%**	0.001	0.970	0.008
	**RT vs RT+HIIT**	9.9 ± 9.1	0.41 ± 0.36	Small		unclear	0.005	0.832	0.064
	**HIIT+RT vs RT+HIIT**	-4.6 ± 4.8	-0.20 ± 0.22	Small	**Possibly ↓**	**@99%**	0.506	0.003	0.121
**Peak Power**	**RT vs HIIT+RT**	11.0 ± 6.2	0.49 ± 0.28	Small	**Very Likely ↑**	**@5/.1%**	< 0.0005	0.954	0.005
	**RT vs RT+HIIT**	9.8 ± 5.2	0.44 ± 0.24	Small	**Likely ↑**	**@5/.1%**	< 0.0005	0.949	0.004
	**HIIT+RT vs RT+HIIT**	-1.0 ± 4.9	-0.05 ± 0.23	Trivial	Likely ↔	@90%	0.131	0.038	0.716

PRE-to-POST within-group and between-group changes in performance variables

For within-group changes (PRE vs POST), and the differences in changes between groups (RT vs HIIT+RT and RT vs RT+HIIT):

p^H value threshold for rejection of harm: < 0.005

p^B value threshold for rejection of benefit: < 0.25

For the differences in changes between HIIT+RT vs RT+HIIT:

$p^{-/+}$ value threshold for rejection of substantially positive (+ve) or negative (-ve) effect: < 0.05

%Δ = mean percent change; %diff = mean percent difference in change scores; SD = standard deviation; ES = standardised effect size (Cohen's d); 90%CI = 90% compatibility interval;
↑= substantially beneficial or positive effect (i.e., improved); ↔ = trivial effect; ↓ = substantially harmful or negative effect (i.e., impaired)
@25/.5% = 25% chance of benefit, 0.5% risk of harm; @5/.1% = 5% chance of beneit, 0.1% risk of harm
@90% = 90% compatibility interval; @99% = 99% compatibility interval
Bold text indicates effects which remained clear at more conservative thresholds after adjusting for multiple inferences.
TBLH = Total Body Less Head

Within-Group Changes (Δ)		Percent Effect %Δ ± SD	Standardised Effect Size (ES) ES (d) ± 90% CL	Magnitude	Likelihood true effect is ↑/↔/↓	Threshold for clear effect:	p^H value (harm)	p^B value (benefit)	p value (NHST)
Leg Press 1-RM	RT	23.9 ± 12.4	0.74 ± 0.29	Moderate	**Most Likely ↑**	**@5/.1%**	< 0.0005	0.998	0.001
	HIIT+RT	28.1 ± 8.3	0.86 ± 0.24	Moderate	**Most Likely ↑**	**@5/.1%**	< 0.0005	1.000	< 0.0005
	RT+HIIT	27.4 ± 7.9	0.84 ± 0.24	Moderate	**Most Likely ↑**	**@5/.1%**	< 0.0005	1.000	< 0.0005
Leg Press 1-RM (relative)	RT	21.0 ± 11.9	0.90 ± 0.37	Moderate	**Most Likely ↑**	**@5/.1%**	< 0.0005	0.997	0.001
	HIIT+RT	25.8 ± 7.8	1.08 ± 0.31	Moderate	**Most Likely ↑**	**@5/.1%**	< 0.0005	1.000	< 0.0005
	RT+HIIT	26.7 ± 6.3	1.11 ± 0.29	Moderate	**Most Likely ↑**	**@5/.1%**	< 0.0005	1.000	< 0.0005
CMJ Height	RT	5.3 ± 6.3	0.32 ± 0.26	Small	Likely ↑	@25/.5%	0.003	0.805	0.046
	HIIT+RT	4.5 ± 6.3	0.27 ± 0.23	Small	Possibly ↑	@25/.5%	0.002	0.710	0.056
	RT+HIIT	-0.1 ± 4.8	0.00 ± 0.17	Trivial	**Likely ↔**	**@5/.1%**	0.033	0.028	0.959
CMJ Velocity	RT	2.2 ± 2.7	0.31 ± 0.25	Small	Likely ↑	@25/.5%	0.002	0.786	0.046
	HIIT+RT	2.2 ± 2.7	0.31 ± 0.23	Small	Likely ↑	@25/.5%	0.001	0.798	0.031
	RT+HIIT	0.2 ± 1.9	0.03 ± 0.16	Trivial	**Likely ↔**	**@5/.1%**	0.014	0.039	0.761
CMJ Force (absolute)	RT	10.1 ± 10.1	0.43 ± 0.29	Small	Likely ↑	@25/.5%	0.002	0.912	0.025
	HIIT+RT	4.0 ± 9.7	0.18 ± 0.25	Trivial		unclear	0.011	0.432	0.220
	RT+HIIT	1.2 ± 9.4	0.05 ± 0.24	Trivial	Likely ↔	@25/.5%	0.043	0.141	0.699
CMJ Force (relative)	RT	6.5 ± 10.7	0.34 ± 0.36	Small		unclear	0.012	0.758	0.111
	HIIT+RT	3.1 ± 9.3	0.16 ± 0.29	Trivial		unclear	0.023	0.414	0.316
	RT+HIIT	0.3 ± 9.9	0.02 ± 0.30	Trivial	Possibly ↔	@25/.5%	0.111	0.144	0.927
CMJ Power (absolute)	RT	9.8 ± 7.6	0.50 ± 0.26	Small	**Very Likely ↑**	**@5/.1%**	< 0.0005	0.969	0.006
	HIIT+RT	4.9 ± 5.8	0.26 ± 0.19	Small	**Possibly ↑**	**@5/.1%**	0.001	0.713	0.027
	RT+HIIT	3.2 ± 4.5	0.17 ± 0.14	Trivial	**Possibly ↑**	**@5/.1%**	< 0.0005	0.361	0.053
CMJ Power (relative)	RT	6.0 ± 6.6	0.39 ± 0.28	Small	Likely ↑	@25/.5%	0.002	0.880	0.028
	HIIT+RT	3.2 ± 6.1	0.21 ± 0.24	Small		unclear	0.006	0.537	0.132
	RT+HIIT	2.6 ± 4.7	0.17 ± 0.18	Trivial	Possibly ↑	@25/.5%	0.002	0.391	0.116
Total Lean Mass (TBLH)	RT	2.8 ± 2.3	0.19 ± 0.11	Trivial	**Possibly ↑**	**@5/.1%**	< 0.0005	0.464	0.009
	HIIT+RT	2.9 ± 1.4	0.20 ± 0.07	Small	**Possibly ↑**	**@5/.1%**	< 0.0005	0.495	< 0.0005
	RT+HIIT	3.9 ± 1.5	0.26 ± 0.08	Small	**Likely ↑**	**@5/.1%**	< 0.0005	0.906	< 0.0005
Upper Lean Mass (TBLH)	RT	2.3 ± 2.1	0.16 ± 0.10	Trivial	**Likely ↔**	**@5/.1%**	< 0.0005	0.241	0.015
	HIIT+RT	2.7 ± 2.4	0.19 ± 0.11	Trivial	**Possibly ↑**	**@5/.1%**	< 0.0005	0.453	0.008
	RT+HIIT	4.1 ± 1.4	0.29 ± 0.09	Small	**Very Likely ↑**	**@5/.1%**	< 0.0005	0.953	< 0.0005
Lower Lean Mass (TBLH)	RT	3.7 ± 3.2	0.23 ± 0.13	Small	**Possibly ↑**	**@5/.1%**	< 0.0005	0.645	0.010
	HIIT+RT	3.3 ± 2.1	0.21 ± 0.09	Small	**Possibly ↑**	**@5/.1%**	< 0.0005	0.555	0.001
	RT+HIIT	3.5 ± 2.4	0.22 ± 0.10	Small	**Possibly ↑**	**@5/.1%**	< 0.0005	0.611	0.002
Total Fat Mass (TBLH)	RT	2.0 ± 6.3	0.05 ± 0.09	Trivial	**Very Likely ↔**	**@5/.1%**	0.006	< 0.0005	0.363
	HIIT+RT	-3.0 ± 11.0	-0.07 ± 0.14	Trivial	**Likely ↔**	**@5/.1%**	0.003	0.065	0.380
	RT+HIIT	-11.0 ± 11.7	-0.27 ± 0.16	Small	**Likely ↑**	**@5/.1%**	< 0.0005	0.779	0.009
Absolute VO2peak	RT	0.7 ± 5.9	0.03 ± 0.19	Trivial	Likely ↔	@25/.5%	0.024	0.068	0.745
	HIIT+RT	10.7 ± 1.9	0.53 ± 0.13	Small	**Most Likely ↑**	**@5/.1%**	< 0.0005	1.000	< 0.0005
	RT+HIIT	8.7 ± 5.0	0.43 ± 0.17	Small	**Very Likely ↑**	**@5/.1%**	< 0.0005	0.985	0.001
Relative VO2peak	RT	-2.1 ± 4.4	-0.13 ± 0.16	Trivial	**Likely ↔**	**@5/.1%**	0.205	0.002	0.170
	HIIT+RT	8.6 ± 3.4	0.48 ± 0.15	Small	**Most Likely ↑**	**@5/.1%**	< 0.0005	0.998	< 0.0005
	RT+HIIT	7.6 ± 4.2	0.43 ± 0.17	Small	**Very Likely ↑**	**@5/.1%**	< 0.0005	0.986	< 0.0005
Lactate Threshold	RT	-2.2 ± 17.1	-0.10 ± 0.42	Trivial	Possibly ↓	@25/.5%	0.331	0.114	0.681
	HIIT+RT	19.6 ± 5.5	0.77 ± 0.21	Moderate	**Most Likely ↑**	**@5/.1%**	< 0.0005	1.000	< 0.0005
	RT+HIIT	16.1 ± 12.8	0.64 ± 0.33	Moderate	**Very Likely ↑**	**@5/.1%**	< 0.0005	0.984	0.004
Peak Power	RT	-1.1 ± 6.7	-0.05 ± 0.19	Trivial	**Likely ↔**	**@5/.1%**	0.088	0.021	0.638
	HIIT+RT	14.5 ± 7.1	0.63 ± 0.23	Moderate	**Most Likely ↑**	**@5/.1%**	< 0.0005	0.998	< 0.0005
	RT+HIIT	13.9 ± 7.5	0.61 ± 0.23	Moderate	**Most Likely ↑**	**@5/.1%**	< 0.0005	0.997	< 0.0005

Between-Group Differences in Δ		Percent Effect %diff ± 90%CL	Standardised Effect Size (ES) ES (d) ± 90% CL	Magnitude	Likelihood true effect is ↑/↔/↓	Threshold for clear effect:	$p^{H/-}$ value (harm/-ve)	$p^{B/+}$ value (bene/+ve)	p value (NHST)
Leg Press 1-RM	RT vs HIIT+RT	3.4 ± 8.5	0.12 ± 0.29	Trivial		unclear	0.036	0.304	0.487
	RT vs RT+HIIT	2.8 ± 8.4	0.09 ± 0.28	Trivial		unclear	0.044	0.260	0.561
	HIIT+RT vs RT+HIIT	-0.6 ± 6.0	-0.02 ± 0.21	Trivial	Likely ↔	@90%	0.078	0.043	0.868
Leg Press 1-RM (relative)	RT vs HIIT+RT	4.0 ± 8.2	0.18 ± 0.37	Trivial		unclear	0.045	0.468	0.395
	RT vs RT+HIIT	4.7 ± 7.9	0.21 ± 0.36	Small		unclear	0.031	0.527	0.303
	HIIT+RT vs RT+HIIT	0.7 ± 5.4	0.03 ± 0.25	Trivial		unclear	0.065	0.128	0.835
CMJ Height	RT vs HIIT+RT	-0.8 ± 5.0	-0.05 ± 0.31	Trivial	Possibly ↔	@25/.5%	0.207	0.089	0.778
	RT vs RT+HIIT	-5.1 ± 4.3	-0.33 ± 0.29	Small	**Likely ↓**	**@5/.1%**	0.776	0.003	0.061
	HIIT+RT vs RT+HIIT	-4.3 ± 4.1	-0.28 ± 0.27	Small	**Possibly ↓**	**@99%**	0.686	0.004	0.089
CMJ Velocity	RT vs HIIT+RT	0.0 ± 2.2	0.00 ± 0.30	Trivial	Possibly ↔	@25/.5%	0.136	0.130	0.989
	RT vs RT+HIIT	-2.0 ± 1.9	-0.28 ± 0.28	Small	**Possibly ↓**	**@5/.1%**	0.699	0.004	0.088
	HIIT+RT vs RT+HIIT	-2.0 ± 1.8	-0.28 ± 0.26	Small	**Possibly ↓**	**@99%**	0.703	0.003	0.073
CMJ Force (absolute)	RT vs HIIT+RT	-5.6 ± 7.4	-0.26 ± 0.35	Small	**Possibly ↓**	**@5/.1%**	0.610	0.019	0.215
	RT vs RT+HIIT	-8.2 ± 7.1	-0.38 ± 0.35	Small	**Likely ↓**	**@5/.1%**	0.809	0.006	0.072
	HIIT+RT vs RT+HIIT	-2.7 ± 7.0	-0.12 ± 0.32	Trivial	Possibly ↓	@90%	0.345	0.049	0.509
CMJ Force (relative)	RT vs HIIT+RT	-3.2 ± 7.7	-0.18 ± 0.42	Trivial	Possibly ↓	@25/.5%	0.463	0.072	0.478
	RT vs RT+HIIT	-5.9 ± 7.6	-0.33 ± 0.43	Small	**Possibly ↓**	**@5/.1%**	0.689	0.027	0.209
	HIIT+RT vs RT+HIIT	-2.7 ± 7.1	-0.15 ± 0.37	Trivial		unclear	0.413	0.070	0.514
CMJ Power (absolute)	RT vs HIIT+RT	-4.4 ± 5.1	-0.24 ± 0.29	Small	**Possibly ↓**	**@5/.1%**	0.604	0.009	0.156
	RT vs RT+HIIT	-6.0 ± 4.7	-0.33 ± 0.28	Small	**Likely ↓**	**@5/.1%**	0.793	0.002	0.048
	HIIT+RT vs RT+HIIT	-1.6 ± 3.9	-0.09 ± 0.22	Trivial	Likely ↔	@90%	0.190	0.016	0.482
CMJ Power (relative)	RT vs HIIT+RT	-2.6 ± 4.8	-0.18 ± 0.34	Small	**Possibly ↓**	**@5/.1%**	0.458	0.034	0.363
	RT vs RT+HIIT	-3.2 ± 4.4	-0.22 ± 0.31	Small	**Possibly ↓**	**@5/.1%**	0.545	0.016	0.226
	HIIT+RT vs RT+HIIT	-0.6 ± 4.1	-0.04 ± 0.28	Trivial		unclear	0.168	0.076	0.802
Total Lean Mass (TBLH)	RT vs HIIT+RT	0.1 ± 1.6	0.01 ± 0.11	Trivial	Very Likely ↔	@25/.5%	0.003	0.004	0.937
	RT vs RT+HIIT	1.0 ± 1.6	0.07 ± 0.11	Trivial	**Very Likely ↔**	**@5/.1%**	0.001	0.032	0.288
	HIIT+RT vs RT+HIIT	0.9 ± 1.1	0.07 ± 0.08	Trivial	**Most Likely ↔**	**@99%**	< 0.0005	0.004	0.159
Upper Lean Mass (TBLH)	RT vs HIIT+RT	0.5 ± 1.8	0.03 ± 0.13	Trivial	Very Likely ↔	@25/.5%	0.003	0.021	0.667
	RT vs RT+HIIT	1.8 ± 1.5	0.13 ± 0.11	Trivial	**Likely ↔**	**@5/.1%**	< 0.0005	0.132	0.056
	HIIT+RT vs RT+HIIT	1.3 ± 1.6	0.09 ± 0.11	Trivial	**Likely ↔**	**@99%**	< 0.0005	0.064	0.164
Lower Lean Mass (TBLH)	RT vs HIIT+RT	-0.3 ± 2.2	-0.02 ± 0.14	Trivial	**Very Likely ↔**	**@5/.1%**	0.019	0.007	0.794
	RT vs RT+HIIT	-0.2 ± 2.3	-0.01 ± 0.14	Trivial	**Very Likely ↔**	**@5/.1%**	0.018	0.011	0.888
	HIIT+RT vs RT+HIIT	0.1 ± 1.7	0.01 ± 0.11	Trivial	**Very Likely ↔**	**@99%**	0.002	0.004	0.887
Total Fat Mass (TBLH)	RT vs HIIT+RT	-4.9 ± 6.5	-0.12 ± 0.16	Trivial	**Likely ↔**	**@5/.1%**	0.002	0.189	0.216
	RT vs RT+HIIT	-12.8 ± 6.3	-0.32 ± 0.18	Small	**Likely ↑**	**@5/.1%**	< 0.0005	0.863	0.005
	HIIT+RT vs RT+HIIT	-8.3 ± 7.7	-0.20 ± 0.20	Small	**Possibly ↑**	**@99%**	0.001	0.499	0.092
Absolute VO2peak	RT vs HIIT+RT	10.0 ± 4.0	0.49 ± 0.21	Small	**Very Likely ↑**	**@5/.1%**	< 0.0005	0.985	0.001
	RT vs RT+HIIT	8.0 ± 4.7	0.40 ± 0.24	Small	**Likely ↑**	**@5/.1%**	< 0.0005	0.917	0.007
	HIIT+RT vs RT+HIIT	-1.8 ± 2.9	-0.09 ± 0.15	Trivial	**Likely ↔**	**@99%**	0.121	0.003	0.297
Relative VO2peak	RT vs HIIT+RT	11.0 ± 3.5	0.61 ± 0.22	Moderate	**Most Likely ↑**	**@5/.1%**	< 0.0005	0.998	< 0.0005
	RT vs RT+HIIT	10.0 ± 3.7	0.56 ± 0.23	Small	**Very Likely ↑**	**@5/.1%**	< 0.0005	0.993	< 0.0005
	HIIT+RT vs RT+HIIT	-0.9 ± 2.9	-0.05 ± 0.17	Trivial	Likely ↔	@90%	0.078	0.011	0.605
Lactate Threshold	RT vs HIIT+RT	22.3 ± 12.4	0.87 ± 0.47	Moderate	**Very Likely ↑**	**@5/.1%**	0.001	0.988	0.005
	RT vs RT+HIIT	18.7 ± 13.6	0.74 ± 0.51	Moderate	Very Likely ↑	@25/.5%	0.003	0.957	0.019
	HIIT+RT vs RT+HIIT	-3.0 ± 7.2	-0.13 ± 0.32	Trivial	Possibly ↓	@90%	0.350	0.046	0.487
Peak Power	RT vs HIIT+RT	15.7 ± 6.2	0.68 ± 0.29	Moderate	**Most Likely ↑**	**@5/.1%**	< 0.0005	0.996	< 0.0005
	RT vs RT+HIIT	15.1 ± 6.4	0.65 ± 0.29	Moderate	**Very Likely ↑**	**@5/.1%**	< 0.0005	0.993	< 0.0005
	HIIT+RT vs RT+HIIT	-0.5 ± 5.5	-0.03 ± 0.26	Trivial		unclear	0.127	0.073	0.867

Internal and external training load data

For between-group differences in training loads:

$p^{-/+}$ value threshold for rejection of substantially positive (+ve) or negative (-ve) effect: < 0.05

%diff = mean percent difference; SD = standard deviation; ES = standardised effect size (Cohen’s d); 90%CI = 90% compatibility interval;
↑= substantially positive; ↔ = trivial effect; ↓ = substantially negative
@90% = 90% compatibility interval; @99% = 99% compatibility interval
Bold text indicates effects which remained clear at more conservative thresholds after adjusting for multiple inferences.

Variable	Group	Week 1 Mean ± SD	Week 2 Mean ± SD	Week 3 Mean ± SD	Week 4 Mean ± SD	Week 5 Mean ± SD	Week 6 Mean ± SD	Week 7 Mean ± SD	Week 8 Mean ± SD	Week 9 Mean ± SD	Weekly Average Mean ± SD	Between-group Comparisons	Percent Effect %diff ± 90%CI	Standardised Effect Size (ES) ES(d) ± 90%CI	Magnitud	Likelihood true effect is ↑/↔/↓	Threshold for clear effect:	p^- value (-ve)	p^+ value (+ve)	p value (NHST)
**Internal Load**																				
Resistance Sessions (AU)	RT	113 ± 48	262 ± 90	262 ± 65	271 ± 76	267 ± 73	417 ± 120	355 ± 99	372 ± 110	365 ± 100	269 ± 100	RT vs HIIT+RT	25 ± 20	0.84 ± 0.60	**Moderate**	***Very Likely* ↑**	***@99%***	0.003	0.961	0.022
	HIIT+RT	137 ± 28	338 ± 93	342 ± 90	350 ± 75	338 ± 90	474 ± 85	438 ± 82	427 ± 71	414 ± 84	337 ± 84	RT vs RT+HIIT	2.4 ± 16	0.09 ± 0.57	Trivial		*unclear*	0.197	0.371	0.792
	RT+HIIT	104 ± 37	265 ± 61	265 ± 62	258 ± 61	282 ± 76	417 ± 94	370 ± 70	392 ± 80	384 ± 79	275 ± 76	HIIT+RT vs RT+HIIT	-18 ± 9.3	-0.76 ± 0.44	**Moderate**	***Very Likely* ↓**	***@99%***	0.980	0.000	0.006
Endurance Sessions (AU)	HIIT+RT	212 ± 59	193 ± 52	215 ± 52	242 ± 75	192 ± 59	236 ± 60	261 ± 66	300 ± 80	210 ± 74	218 ± 71									
	RT+HIIT	179 ± 36	171 ± 41	226 ± 48	238 ± 48	188 ± 44	256 ± 50	271 ± 52	306 ± 65	212 ± 51	217 ± 49	HIIT+RT vs RT+HIIT	-0.5 ± 13	-0.02 ± 0.54	Trivial		*unclear*	0.285	0.243	0.949
**External Load**																				
RES - Absolute Volume Load (kg)	RT	14,300 ± 4,100	14,100 ± 3,500	14,300 ± 3,000	12,900 ± 2,600	11,400 ± 2,800	20,800 ± 5,400	19,700 ± 4,700	16,900 ± 4,400	15,200 ± 3,100	14,800 ± 3,900	RT vs HIIT+RT	-4.3 ± 19	-0.18 ± 0.79	Trivial		*unclear*	0.479	0.211	0.706
	HIIT+RT	13,800 ± 3,800	13,600 ± 4,100	13,500 ± 3,700	12,500 ± 3,200	11,700 ± 3,500	20,100 ± 6,100	19,900 ± 5,300	16,700 ± 4,600	14,700 ± 3,100	14,200 ± 4,500	RT vs RT+HIIT	-1.5 ± 17	-0.06 ± 0.67	Trivial		*unclear*	0.364	0.258	0.880
	RT+HIIT	13,800 ± 3,700	13,800 ± 3,300	14,000 ± 3,000	12,700 ± 2,600	11,200 ± 2,700	20,100 ± 4,700	19,600 ± 4,000	16,200 ± 3,100	13,900 ± 2,100	14,600 ± 3,400	HIIT+RT vs RT+HIIT	2.9 ± 19	0.11 ± 0.74	Trivial		*unclear*	0.236	0.423	0.792
END - Absolute Work Done (kJ)	HIIT+RT	913 ± 180	838 ± 190	954 ± 200	1,100 ± 220	910 ± 190	1,060 ± 210	1,180 ± 240	1,310 ± 250	1,020 ± 230	997 ± 230									
	RT+HIIT	846 ± 180	786 ± 180	887 ± 200	1,020 ± 210	809 ± 180	988 ± 200	1,100 ± 210	1,220 ± 240	962 ± 220	929 ± 190	HIIT+RT vs RT+HIIT	-6.7 ± 14	-0.34 ± 0.74	Small		*unclear*	0.631	0.108	0.425

Readiness-to-train questionnaire data

For between-group differences:

$p^{-/+}$ value threshold for rejection of substantially positive (+ve) or negative (-ve) effect: < 0.05

SD = standard deviation; ES = standardised effect size (Cohen's d); 90%CI = 90% compatibility interval;
↑= substantially positive; ↔ = trivial effect; ↓ = substantially negative
@90% = 90% compatibility interval; @99% = 99% compatibility interval
Bold text indicates effects which remained clear at more conservative thresholds after adjusting for multiple inferences.

Variable	Group	Week 1 Mean ± SD	Week 2 Mean ± SD	Week 3 Mean ± SD	Week 4 Mean ± SD	Week 5 Mean ± SD	Week 6 Mean ± SD	Week 7 Mean ± SD	Week 8 Mean ± SD	Week 9 Mean ± SD	Weekly Average Mean ± SD	Between-group Comparisons	Difference (raw) mean ± 90%CI	Standardised Effect Size (ES) ES(d) ± 90%CI	Magnitud	Likelihood true effect is ↑/↔/↓	Threshold for clear effect:	p^- value (-ve)	p^+ value (+ve)	p value (NHST)
**Resistance Sessions**																				
Total Score (/25)	RT	18.0 ± *3.2*	18.6 ± *2.2*	18.1 ± *2.9*	18.8 ± *2.5*	18.1 ± *2.9*	17.6 ± *2.4*	18.2 ± *2.5*	17.4 ± *2.8*	18.3 ± *2.3*	18.1 ± *2.7*	RT vs HIIT+RT	-1.8 ± *1.4*	-0.60 ± *0.46*	**Moderate**	***Likely ↓***	*@99%*	0.928	0.003	0.031
	HIIT+RT	17.0 ± *2.8*	16.6 ± *2.9*	15.8 ± *3.5*	16.5 ± *3.1*	16.4 ± *2.6*	15.9 ± *2.6*	16.6 ± *2.3*	16.2 ± *2.4*	15.7 ± *2.8*	16.3 ± *2.9*	RT vs RT+HIIT	-0.6 ± *1.8*	-0.20 ± *0.60*	Small		*unclear*	0.502	0.131	0.569
	RT+HIIT	17.9 ± *2.8*	17.3 ± *3.6*	18.4 ± *3.3*	17.7 ± *3.2*	17.8 ± *3.7*	17.1 ± *3.3*	17.2 ± *3.3*	17.1 ± *3.6*	17.7 ± *3.2*	17.5 ± *3.4*	HIIT+RT vs RT+HIIT	1.2 ± *1.9*	0.40 ± *0.62*	Small		*unclear*	0.055	0.709	0.277
Fatigue	RT	3.3 ± *1.0*	3.3 ± *0.7*	3.3 ± *0.8*	3.5 ± *0.8*	3.2 ± *0.9*	3.2 ± *0.8*	3.5 ± *0.6*	3.2 ± *0.9*	3.3 ± *0.7*	3.3 ± *0.8*	RT vs HIIT+RT	-0.3 ± *0.4*	-0.36 ± *0.43*	Small	*Possibly ↓*	*@90%*	0.731	0.019	0.168
	HIIT+RT	3.1 ± *0.9*	3.0 ± *0.9*	2.9 ± *0.9*	3.0 ± *0.8*	3.2 ± *0.6*	2.9 ± *0.8*	2.9 ± *0.7*	3.1 ± *0.8*	2.9 ± *0.8*	3.0 ± *0.8*	RT vs RT+HIIT	-0.1 ± *0.5*	-0.10 ± *0.55*	Trivial		*unclear*	0.380	0.182	0.760
	RT+HIIT	3.5 ± *0.9*	3.3 ± *0.9*	3.3 ± *1.0*	3.3 ± *0.8*	3.3 ± *1.0*	3.1 ± *0.9*	3.1 ± *1.0*	3.0 ± *1.0*	3.1 ± *0.9*	3.2 ± *1.0*	HIIT+RT vs RT+HIIT	0.2 ± *0.5*	0.26 ± *0.56*	Small		*unclear*	0.089	0.570	0.441
Sleep Quality	RT	3.5 ± *0.8*	3.7 ± *0.7*	3.6 ± *0.8*	3.7 ± *0.7*	3.7 ± *0.7*	3.7 ± *0.8*	3.6 ± *0.7*	3.4 ± *0.8*	3.6 ± *0.8*	3.6 ± *0.8*	RT vs HIIT+RT	-0.2 ± *0.3*	-0.21 ± *0.32*	Small	*Possibly ↓*	*@90%*	0.511	0.019	0.280
	HIIT+RT	3.5 ± *0.8*	3.4 ± *0.9*	3.2 ± *0.8*	3.5 ± *0.9*	3.3 ± *0.8*	3.5 ± *0.8*	3.6 ± *0.9*	3.6 ± *0.7*	3.3 ± *0.7*	3.4 ± *0.8*	RT vs RT+HIIT	-0.1 ± *0.4*	-0.08 ± *0.44*	Trivial		*unclear*	0.326	0.139	0.747
	RT+HIIT	3.7 ± *0.8*	3.6 ± *0.9*	3.8 ± *0.7*	3.5 ± *0.9*	3.6 ± *1.0*	3.4 ± *0.9*	3.4 ± *0.9*	3.5 ± *0.9*	3.5 ± *1.0*	3.5 ± *0.9*	HIIT+RT vs RT+HIIT	0.1 ± *0.4*	0.12 ± *0.46*	Trivial		*unclear*	0.121	0.387	0.654
General Muscle Soreness	RT	3.1 ± *1.3*	3.4 ± *1.0*	3.6 ± *0.8*	3.7 ± *0.8*	3.5 ± *1.0*	3.1 ± *0.8*	3.4 ± *0.8*	3.4 ± *1.0*	3.7 ± *0.6*	3.4 ± *0.9*	RT vs HIIT+RT	-0.6 ± *0.4*	-0.62 ± *0.46*	**Small**	***Likely ↓***	*@99%*	0.931	0.003	0.030
	HIIT+RT	2.7 ± *1.1*	3.1 ± *0.9*	3.1 ± *0.8*	2.9 ± *0.9*	3.1 ± *0.7*	2.7 ± *0.9*	3.0 ± *0.7*	2.6 ± *0.7*	2.7 ± *0.8*	2.9 ± *0.8*	RT vs RT+HIIT	-0.3 ± *0.5*	-0.37 ± *0.55*	Small	*Possibly ↓*	*@90%*	0.693	0.046	0.267
	RT+HIIT	2.9 ± *0.9*	2.9 ± *0.9*	3.4 ± *1.0*	3.1 ± *0.9*	3.3 ± *1.0*	3.2 ± *0.8*	3.0 ± *1.0*	3.1 ± *1.1*	3.1 ± *1.1*	3.1 ± *1.0*	HIIT+RT vs RT+HIIT	0.2 ± *0.5*	0.25 ± *0.53*	Small		*unclear*	0.081	0.564	0.430
Stress	RT	3.9 ± *0.7*	4.0 ± *0.6*	3.8 ± *0.6*	3.8 ± *0.5*	3.8 ± *0.7*	3.7 ± *0.4*	3.7 ± *0.6*	3.5 ± *0.7*	3.7 ± *0.6*	3.8 ± *0.6*	RT vs HIIT+RT	-0.4 ± *0.3*	-0.51 ± *0.40*	**Small**	***Likely ↓***	*@99%*	0.902	0.003	0.036
	HIIT+RT	3.7 ± *0.7*	3.4 ± *0.8*	3.2 ± *0.9*	3.5 ± *0.7*	3.3 ± *0.8*	3.3 ± *0.8*	3.5 ± *0.7*	3.4 ± *0.7*	3.3 ± *0.8*	3.4 ± *0.8*	RT vs RT+HIIT	-0.1 ± *0.3*	-0.14 ± *0.46*	Trivial		*unclear*	0.412	0.109	0.607
	RT+HIIT	3.8 ± *0.6*	3.6 ± *0.8*	3.8 ± *0.7*	3.8 ± *0.7*	3.7 ± *0.7*	3.5 ± *0.7*	3.6 ± *0.7*	3.5 ± *0.8*	3.8 ± *0.6*	3.7 ± *0.7*	HIIT+RT vs RT+HIIT	0.3 ± *0.4*	0.37 ± *0.57*	Small		*unclear*	0.051	0.690	0.281
Mood	RT	4.1 ± *0.6*	4.3 ± *0.5*	3.9 ± *0.6*	4.0 ± *0.5*	4.0 ± *0.5*	3.9 ± *0.6*	4.0 ± *0.5*	3.9 ± *0.4*	4.0 ± *0.3*	4.0 ± *0.5*	RT vs HIIT+RT	-0.4 ± *0.2*	-0.68 ± *0.39*	**Moderate**	***Very Likely ↓***	*@99%*	0.976	0.000	0.006
	HIIT+RT	3.9 ± *0.6*	3.7 ± *0.6*	3.4 ± *1.1*	3.7 ± *0.7*	3.5 ± *0.7*	3.6 ± *0.7*	3.6 ± *0.6*	3.5 ± *0.5*	3.5 ± *0.8*	3.6 ± *0.7*	RT vs RT+HIIT	0.0 ± *0.3*	-0.03 ± *0.42*	Trivial		*unclear*	0.246	0.179	0.905
	RT+HIIT	4.2 ± *0.4*	4.0 ± *0.7*	4.1 ± *0.5*	4.0 ± *0.5*	4.0 ± *0.7*	3.8 ± *0.7*	4.0 ± *0.5*	4.0 ± *0.5*	4.1 ± *0.5*	4.0 ± *0.6*	HIIT+RT vs RT+HIIT	0.4 ± *0.3*	0.65 ± *0.47*	**Moderate**	***Likely ↑***	*@99%*	0.002	0.942	0.026
**Endurance Sessions**																				
Total Score (/25)	HIIT+RT	17.8 ± *2.9*	16.7 ± *3.2*	16.3 ± *3.3*	17.6 ± *2.8*	16.7 ± *2.9*	17.3 ± *2.8*	18.7 ± *3.1*	17.2 ± *3.2*	16.2 ± *2.8*	17.1 ± *3.1*									
	RT+HIIT	17.6 ± *2.6*	17.1 ± *3.2*	17.9 ± *3.1*	17.5 ± *3.2*	17.7 ± *3.6*	17.0 ± *3.3*	17.0 ± *3.4*	17.2 ± *3.6*	17.7 ± *3.1*	17.3 ± *3.3*	HIIT+RT vs RT+HIIT	0.2 ± *1.9*	0.06 ± *0.57*	Trivial		*unclear*	0.221	0.336	0.861
Fatigue	HIIT+RT	3.2 ± *0.9*	3.1 ± *1.0*	3.1 ± *1.0*	3.4 ± *0.7*	3.2 ± *0.9*	3.2 ± *0.9*	3.6 ± *0.9*	3.2 ± *1.1*	3.0 ± *0.8*	3.2 ± *0.9*									
	RT+HIIT	3.2 ± *0.9*	3.2 ± *0.9*	3.3 ± *1.0*	3.2 ± *0.9*	3.3 ± *1.0*	3.2 ± *1.0*	3.2 ± *1.0*	3.2 ± *0.9*	3.2 ± *0.8*	3.2 ± *0.9*	HIIT+RT vs RT+HIIT	0.0 ± *0.5*	-0.01 ± *0.54*	Trivial		*unclear*	0.268	0.258	0.987
Sleep Quality	HIIT+RT	3.6 ± *0.8*	3.4 ± *0.9*	3.2 ± *0.8*	3.5 ± *0.8*	3.3 ± *0.9*	3.5 ± *0.8*	3.7 ± *0.9*	3.5 ± *0.9*	3.2 ± *0.8*	3.4 ± *0.9*									
	RT+HIIT	3.7 ± *0.9*	3.5 ± *0.9*	3.7 ± *0.7*	3.5 ± *0.8*	3.6 ± *0.9*	3.5 ± *0.8*	3.5 ± *0.9*	3.5 ± *1.0*	3.5 ± *0.9*	3.5 ± *0.9*	HIIT+RT vs RT+HIIT	0.1 ± *0.4*	0.10 ± *0.44*	Trivial		*unclear*	0.130	0.343	0.711
General Muscle Soreness	HIIT+RT	3.2 ± *1.2*	3.2 ± *1.0*	3.3 ± *0.8*	3.4 ± *0.9*	3.3 ± *0.8*	3.1 ± *1.1*	3.7 ± *0.9*	3.3 ± *0.9*	3.1 ± *0.8*	3.3 ± *0.9*									
	RT+HIIT	2.8 ± *0.8*	2.8 ± *0.7*	3.3 ± *0.8*	3.1 ± *0.8*	3.1 ± *1.0*	2.9 ± *0.9*	3.0 ± *0.9*	3.0 ± *1.2*	3.0 ± *0.9*	3.0 ± *0.9*	HIIT+RT vs RT+HIIT	-0.3 ± *0.5*	-0.32 ± *0.52*	Small	*Possibly ↓*	*@90%*	0.653	0.049	0.297
Stress	HIIT+RT	3.7 ± *0.7*	3.4 ± *0.8*	3.2 ± *1.0*	3.4 ± *0.8*	3.3 ± *0.8*	3.6 ± *0.7*	3.8 ± *0.6*	3.6 ± *0.9*	3.4 ± *0.8*	3.5 ± *0.8*									
	RT+HIIT	3.8 ± *0.6*	3.6 ± *0.8*	3.6 ± *0.7*	3.7 ± *0.7*	3.7 ± *0.8*	3.6 ± *0.7*	3.5 ± *0.7*	3.7 ± *0.7*	3.9 ± *0.6*	3.7 ± *0.7*	HIIT+RT vs RT+HIIT	0.2 ± *0.4*	0.21 ± *0.51*	Small		*unclear*	0.091	0.514	0.486
Mood	HIIT+RT	4.1 ± *0.6*	3.7 ± *0.7*	3.4 ± *1.0*	3.9 ± *0.6*	3.6 ± *0.7*	3.8 ± *0.6*	4.0 ± *0.6*	3.5 ± *0.9*	3.5 ± *0.7*	3.7 ± *0.7*									
	RT+HIIT	4.1 ± *0.3*	4.0 ± *0.6*	3.9 ± *0.5*	4.0 ± *0.6*	4.0 ± *0.7*	3.8 ± *0.7*	3.8 ± *0.6*	3.9 ± *0.6*	4.0 ± *0.6*	3.9 ± *0.6*	HIIT+RT vs RT+HIIT	0.2 ± *0.3*	0.35 ± *0.45*	Small	*Possibly ↑*	*@90%*	0.023	0.719	0.187

Habitual dietary intake data

	Resistance-Only (RT)			HIIT+RT			RT+HIIT		
	Mean		*SD*	Mean		*SD*	Mean		*SD*
**Absolute**									
Energy Intake ($kcal \cdot d^{-1}$)	**2457**	±	*519*	**2649**	±	*617*	**2691**	±	*703*
Carbohydrate ($g \cdot d^{-1}$)	**235**	±	*87*	**285**	±	*97*	**266**	±	*71*
Protein ($g \cdot d^{-1}$)	**153**	±	*56*	**137**	±	*34*	**156**	±	*45*
Fat ($g \cdot kg^{-1}$)	**88**	±	*24*	**110**	±	*41*	**100**	±	*32*
**Relative to body mass**									
Energy Intake ($kcal \cdot kg \cdot day^{-1}$)	**33**	±	*8*	**36**	±	*11*	**36**	±	*9*
Carbohydrate ($g \cdot kg \cdot day^{-1}$)	**3.2**	±	*1.3*	**4.0**	±	*1.9*	**3.6**	±	*1.0*
Protein ($g \cdot kg \cdot day^{-1}$)	**2.0**	±	*0.7*	**1.9**	±	*0.6*	**2.1**	±	*0.6*
Fat ($g \cdot kg \cdot day^{-1}$)	**1.2**	±	*0.4*	**1.5**	±	*0.4*	**1.4**	±	*0.4*

Between Group Differences at Baseline		Effect Size	Magnitude
**Absolute**			
Energy Intake	**RT vs HIIT+RT**	0.26	Small
	**RT vs RT+HIIT**	0.30	Small
	**HIIT+RT vs RT+HIIT**	-0.04	Trivial
Carbohydrate	**RT vs HIIT+RT**	0.58	Small
	**RT vs RT+HIIT**	0.44	Small
	**HIIT+RT vs RT+HIIT**	0.14	Trivial
Protein	**RT vs HIIT+RT**	-0.25	Small
	**RT vs RT+HIIT**	0.15	Trivial
	**HIIT+RT vs RT+HIIT**	-0.40	Small
Fat	**RT vs HIIT+RT**	0.51	Small
	**RT vs RT+HIIT**	0.28	Small
	**HIIT+RT vs RT+HIIT**	0.23	Small
**Relative to body mass**			
Energy Intake	**RT vs HIIT+RT**	0.28	Small
	**RT vs RT+HIIT**	0.34	Small
	**HIIT+RT vs RT+HIIT**	-0.06	Trivial
Carbohydrate	**RT vs HIIT+RT**	0.54	Small
	**RT vs RT+HIIT**	0.44	Small
	**HIIT+RT vs RT+HIIT**	0.11	Trivial
Protein	**RT vs HIIT+RT**	-0.20	Small
	**RT vs RT+HIIT**	0.20	Small
	**HIIT+RT vs RT+HIIT**	-0.40	Small
Fat	**RT vs HIIT+RT**	0.55	Small
	**RT vs RT+HIIT**	0.34	Small
	**HIIT+RT vs RT+HIIT**	0.21	Small
