# Supplementary figures and images for: Order of same-day concurrent training influences some indices of power development, but not strength, lean mass, or aerobic fitness in healthy, moderately-active men after 9 weeks of training
Source: PLoS One. 2020 May 14;15(5):e0233134. doi: 10.1371/journal.pone.0233134 (PMC7224562; doi:10.1371/journal.pone.0233134)

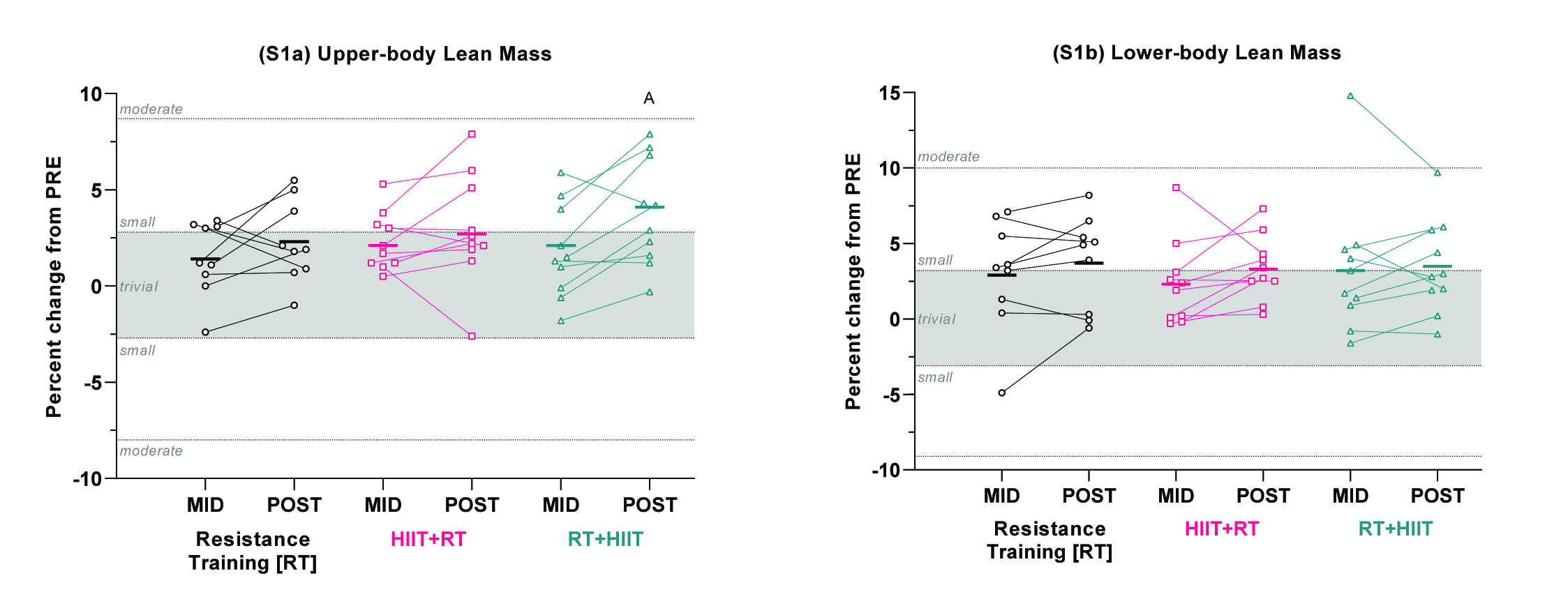

Supplement: S1 Fig — (TIF) [file pone.0233134.s005.tif]

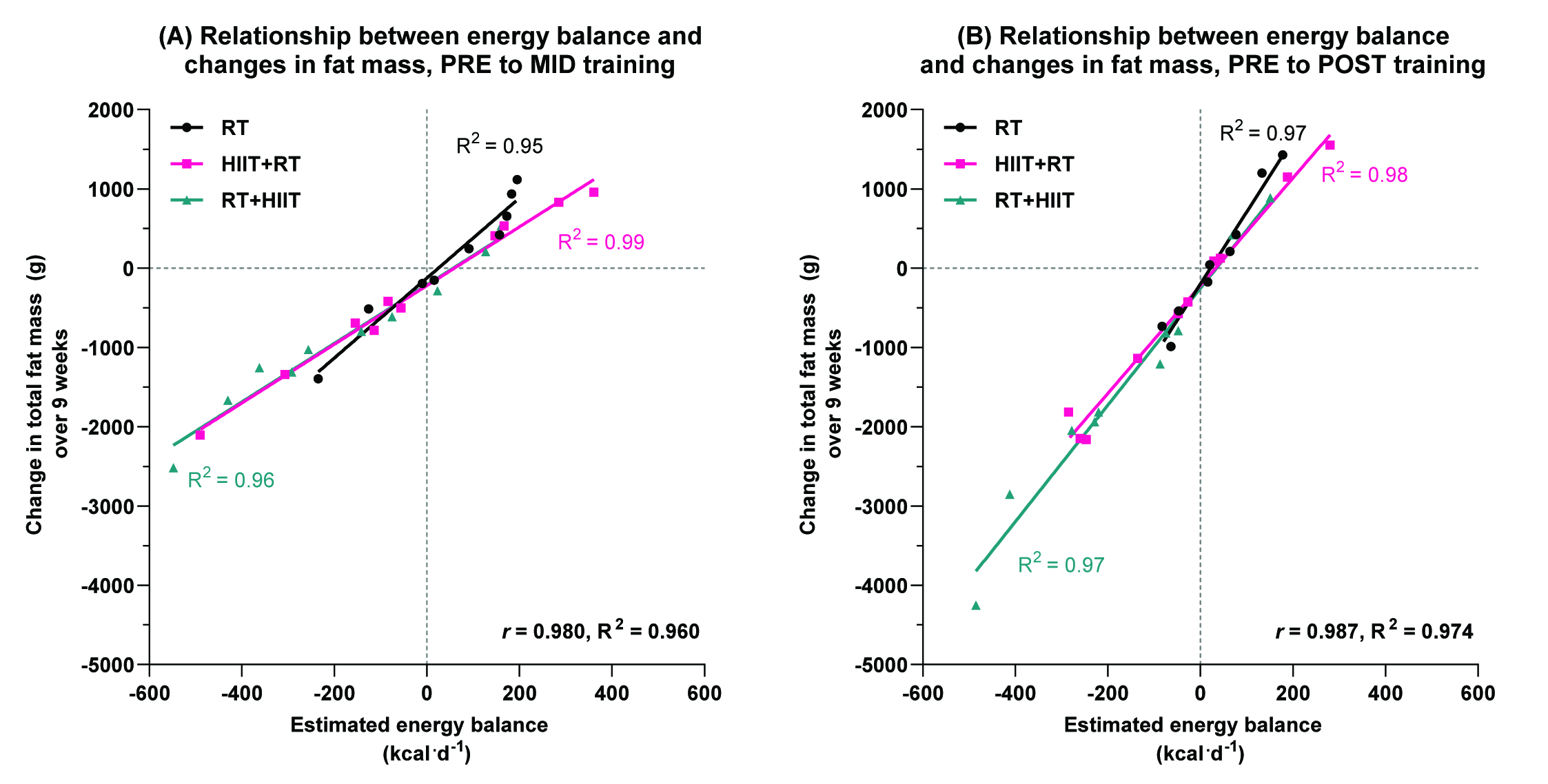

Supplement: S2 Fig — (TIF) [file pone.0233134.s006.tif]
